# Matching events and activities by integrating behavioral aspects and label analysis

**DOI:** 10.1007/s10270-017-0603-z

**Published:** 2017-05-29

**Authors:** Thomas Baier, Claudio Di Ciccio, Jan Mendling, Mathias Weske

**Affiliations:** 1 Lana Labs GmbH, Engeldamm 62, 10179 Berlin, Germany; 2 Wirtschaftsuniversität Wien, Welthandelsplatz 1, 1020 Vienna, Austria; 3 Hasso Plattner Institute, University of Potsdam, Prof.-Dr.-Helmert-Str. 2-3, 14482 Potsdam, Germany

**Keywords:** Process mining, Event mapping, Business process intelligence, Constraint satisfaction, Declare, Natural language processing

## Abstract

Nowadays, business processes are increasingly supported by IT services that produce massive amounts of event data during the execution of a process. These event data can be used to analyze the process using process mining techniques to discover the real process, measure conformance to a given process model, or to enhance existing models with performance information. Mapping the produced events to activities of a given process model is essential for conformance checking, annotation and understanding of process mining results. In order to accomplish this mapping with low manual effort, we developed a semi-automatic approach that maps events to activities using insights from behavioral analysis and label analysis. The approach extracts Declare constraints from both the log and the model to build matching constraints to efficiently reduce the number of possible mappings. These mappings are further reduced using techniques from natural language processing, which allow for a matching based on labels and external knowledge sources. The evaluation with synthetic and real-life data demonstrates the effectiveness of the approach and its robustness toward non-conforming execution logs.

## Introduction

Organizations often support the execution of business processes with IT systems that log each step of participants or systems. Individual entries in such logs represent the execution of services, the submission of a form, or other related tasks that in combination realize a business process. To improve business processes and to align IT process execution with existing business goals, a precise understanding of processes execution is necessary. Using the event data logged by IT systems, process mining techniques help organizations to have a more profound awareness of their processes, in terms of discovering and enhancing process models, or checking the conformance of the execution to the specification [52]. Yet, these process mining techniques face an important challenge: the mapping of log entries produced by IT systems to the corresponding process activities in the process models has to be known. A discovered process model can only be fully understood when the presented results use the terminology that is known to the business analysts. It is indeed a common assumption to rely on prior knowledge of the exact mapping of events to activities. Unfortunately, such abstraction is very often not reflected in reality [43]. Among the other motives, such a mapping is often not existing because (i) the logging mechanism of IT systems captures fine-granular steps on a technical level and (ii) the way in which events are recorded is rarely customizable, especially with legacy systems.

**Fig. 1:**
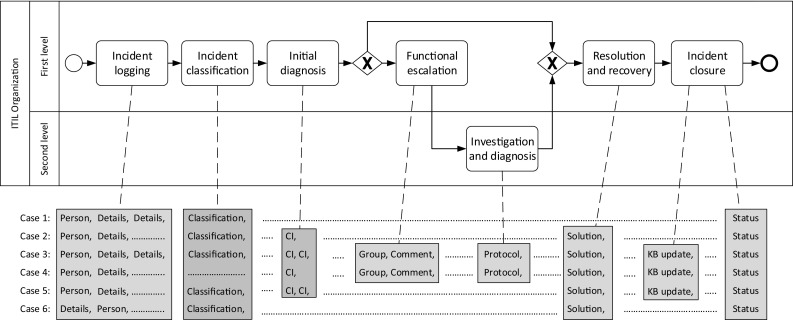
Process model of an incident process modeled in BPMN with links to the execution data

In this paper, we offer means to help the analyst to identify the mapping between a process model and events in an event log in a semi-automated fashion. Defining such a mapping is generally hard to do manually due to its combinatorial complexity. The approach presented in this paper leverages insights from behavioral constraints and linguistic analysis to overcome this complexity. We therefore build on previous work from [5] and [3], which we extend and for which we provide a novel integration mechanism. This allows us to substantially reduce the effort for an analyst. Our approach also informs research into Declare, as it has been mainly used for the modeling of discovered processes from event logs [18, 40]. More specifically, we devise techniques to derive Declare constraints from an existing imperative process model in order to reason about possible matches between events and activities based on the comparison of Declare constraints inferred from the event log and the process model.

In this article, we extend our paper [3] in both the methodology and the evaluation. The Declare-based matching approach is indeed extended with a label analysis based on natural language processing. Furthermore, alternative, relaxed constraints are now included in the framework, and a comparative analysis of the influence of different constraints on the result is reported. A case study based on real-life data is also described for evaluating the integrated approach, along with an in-depth validation of the Declare-based matching approach in settings where events and activities are in both one-to-one and one-to-many relationships.

The remainder of this paper is structured as follows. Section 2 starts by further illustrating the problem with an example and stating the formal definition of the mapping problem and the required formal concepts. Having laid the foundations, the integrated matching technique is introduced in Sect. 3. In Sect. 4, we first validate our Declare-based matching using an industry process model collection and simulated event logs. Second, we evaluate the integrated approach on real-life data from an industry case study. Related work is discussed in Sects. 5 and 6 concludes the work.

## Problem statement and preliminaries

In this section, we motivate our research by the help of an illustrating example. We then revisit preliminary concepts of imperative and declarative process modeling languages. Finally, we discuss the life-cycle of activities.

### Motivating example

Figure 1 depicts the process of incident management based on the definition found in the IT Infrastructure Library (ITIL) [9]. The process is executed by two different roles. The main role is the first level, which is responsible for logging, classifying and initial diagnosis of an incident. In case a first-level agent cannot resolve the incident on their own, the incident can be functionally escalated to a second-level agent. In any case, the first level performs the final resolution and recovery and closes the incident.

Table 1 provides further details on the activities contained in the process model in Fig. 1. Such descriptions are often attached in process modeling tools or separately provided in more detailed work instructions. The goal of these descriptions is to give a better understanding of how the tasks need to be carried out. While our exemplifying descriptions are rather short, these textual instructions can be very long and comprehensive in practical settings.

**Table 1:** Activity descriptions for the incident process

Activity	Description
Incident logging	The first-level agent needs to log the details of the incident and assign the affected person
Incident classification	Depending on the logged details, the appropriate classification needs to be chosen
Initial diagnosis	The assigned first-level supporter needs to search through the Configuration Management Database (CMDB) for the described problem and has to detect the configuration item (CI) that needs fixing
Functional escalation	If no solution can be found, the first-level supporter has to route the incident ticket to the responsible second-level group
Investigation and diagnosis	A second-level supporter needs to perform a technical investigation and diagnosis of the reported incident. The solution is reported back to the first-level group in a protocol entry
Resolution and recovery	Once the solution for the incident is found, it needs to be logged. If required, the customer is informed
Incident closure	If a new solution has been found, the first level supported needs to check whether it may be reused later. If this is the case, the solution needs to be entered into the knowledge base (KB). Finally, the incident is closed

**Table 2:** Event class names

Abbreviation	Complete event name
Person	Person added
Details	Details logged
Classification	Classification specified
CI	CI selected
Group	Group changed
Comment	New comment created
Protocol	New protocol created
Solution	Solution assigned
KB update	KB update performed
Status	Status changed

Aside of the process model, Fig. 1 depicts an excerpt of an event log with six traces. Abbreviations for the event classes are used in the figure, for the sake of readability. For example, $CI$ and $Solution$ stand for “CI selected” and “Solution assigned”, respectively. The complete mapping of the abbreviations to the full names is reported in Table 2. In the remainder, we will use interchangeably the abbreviated or extended version of the labels. The relation between events and activities cannot be easily identified using simple string matching, as the terms used in the event log only rarely occur in the names of the activities. For instance, the two event classes “Person added” and “Details given” have to be related to the activity “Incident logging”. Again, there are sometimes multiple event classes assigned to some of the activities. These may be related to life-cycle transitions of those activities and thereby enable performance analysis for these activities. For example, it may be the case that the first occurring event instance of the two event classes “Person added” and “Details changed” marks the start of the activity “Incident logging” while the last occurrence signals the end. As events are typically recorded with a timestamp, we can calculate the duration of the activity “Incident logging” for each case.

The connection of events to the existing model activities furthermore allows for conformance analysis of the execution data with respect to the defined model behavior. Conformance analysis of the given example reveals that the activity “Initial diagnosis” has been skipped in cases 1 and 6. It could be due either to a fault of the software system, which did not record the associated events, or to a non-compliant enactment of the process by the involved actors. Moreover, the resolution and recovery may not have been correctly executed in case 1, as there is no documented solution. This information can be of high value for the improvement of the process and may be even more important in situations where the execution of certain activities is required by law.

For models like the one shown in Fig. 1, there are different formalizations that we discuss in the following. All formalizations have in common that they specify a process model $$M $$ as a tuple containing among others a set of process activities, which we denote as *A*.

An IT system that supports process executions typically records events for each process instance in an event log [52]. Note that the relation of event instances to process instances might not be trivial in every practical setting. There exist approaches relating event instances to process instances that use event correlation (see [44]). In this work, we therefore assume that the process instance for each event is given. We abstract events as symbols of an alphabet $E$, which is often referred to as the set of event classes. The set of all finite sequences of events is denoted as $E ^*$. Each process instance is represented as a sequence of events and also referred to as trace $t\in E ^*$. For example, $\left[ o,p,o,q \right]$ is a trace with four consecutive events and three different event classes, $o, p, q \in E$. An event log *L* is a multiset of traces.

Confronted with a process model $$M $$ and an event log $$L $$, the challenge is to derive the mapping relation between the activities $$a\in { A}$$ and the event classes $$e \in E $$. In this paper, we assume a 1:N relation as events are typically on a more fine-granular level than activities [56]. Thus, we are looking for the surjective function $$Map: E \rightarrow { A}$$ that maps event classes to their corresponding activities.

In the following subsections, we discuss two paradigms for modeling business processes more in detail, namely the imperative one and the declarative one, and the modeling of activity life cycles. Imperative and declarative approaches depict the behavior of processes from two opposite perspectives. The imperative modeling approach specifies the allowed execution paths for process instances in a temporal structure. Therefore, behavioral relations between pairs of activities often remain implicit. For instance, the activity “Investigation and diagnosis” in Fig. 1 can be executed only eventually after “Incident classification”. This information can be derived by checking the unfoldings of the process model, although it is not explicitly described. On the contrary, the declarative modeling approach only specifies the conditions under which activities can (or cannot) be executed, by means of constraints exerted on single activities and sets of activities. Behavioral relations are thus explicitly modeled, whereas the allowed sequences of activities enactments must be derived by further reasoning on the interplay of the constraints. A declarative model of Fig. 1 would, e.g., represent that a $Precedence$ constraint holds true between “Incident classification” and “Investigation and diagnosis”, but an explicit representation of the in-between sequence flows would be missing.

### Imperative modeling of processes

An imperative process model can formally be defined as a tuple $M = \left\langle { A}, G, T \right\rangle$, where ${ A}$ is a non-empty set of activities, *G* is a set of control nodes, and $T \subseteq ({ A}\cup G) \times ({ A}\cup G)$ is the flow relation, which connects activities and control nodes to build a directed graph. In this paper, we consider the core elements of Business Process Model and Notation (BPMN) [24] to model imperative process models in Fig. 1. BPMN is a standard notation for modeling processes, defined by the Object Management Group (OMG).^1^ Activities are denoted as rounded boxes connected by sequence flows (solid arcs). Control nodes in BPMN include the so-called *gateways*, which are modeled as diamond shapes that split and join control flows into branches. The XOR gateway ($\times$) models the exclusiveness of the following execution branches. In Fig. 1, e.g., the XOR gateway is used to specify that activities “Functional escalation” and “Investigation and diagnosis” can be skipped during the enactment of the process. The AND gateway ($+$) depicts concurrency, i.e., the parallel execution of the branched flow. Information artefacts are depicted as sheets with the top-right corner folded. The exchange of such artefacts as inputs and outputs for activities is depicted by means of dotted arcs. A complete formalization and description of the BPMN notation is out of scope for this paper. We refer the reader to [24, 58] for a comprehensive introduction to BPMN.

### Declarative modeling of processes

Having a process model and an event log, the approach presented in this paper will use Declare to describe their behavior. Declare [53] is natively a declarative process modeling language. It represents workflows by means of temporal rules.^2^ Such rules are meant to impose specific conditions on the execution of activities in process instances. The rationale is that every behavior in the process enactment is allowed as long as it does not violate the specified rules. Due to this, declarative models are said to be “open” in contrast with the “closed” fashion of classical procedural models [40]. Declare rules depict the interplay of every task in the process with the rest of the activities. As a consequence, the behavioral relationships that hold among activities can be analyzed with a local focus on every single activity [32], as a projection of the whole process behavior on a single element of it. The rules pertaining a single task can thus be seen as the task’s footprint in the global behavior of the process. This characteristic allows us to conduct a comparative behavioral analysis within the local scope of activities in the model on the one hand, and events in the log on the other hand. In contrast, imperative models do not consent to separate the local perspective on an activity from the global behavior. This motivates our choice of the Declare modeling language.

The Declare standard provides a predefined library of templates, listing default restrictions that can be imposed on the process control-flow. In particular, Declare rules are exerted on the execution of activities. In this paper, we consider a subset of the full Declare specification that restrict the enactment of one or two activities, as in [20, 39]. For instance, $$ Participation ( a )$$ is a Declare rule expressed on activity $$ a \in { A}$$. It states that $$ a $$ must be carried out in every process instance. Given the activities $$ a , b \in { A}$$, $$ RespondedExistence ( a , b )$$ constrains $$ a $$ and $$ b $$, and imposes that if $$ a $$ is carried out, also $$ b $$ must be carried out at some point during the process instance execution. $$ Participation ( a )$$ expresses a condition on the execution of a single activity. It is thus said to be an *existence rule*, as opposed to *relation rules*, such as $$ RespondedExistence ( a , b )$$, which constrains pairs of activities. In the following, existence templates will be denoted as $$\mathcal {C}_E$$, and $$\mathcal {C}_E( a )$$ is the rule that applies template $$\mathcal {C}_E$$ to activity $$ a \in { A}$$. Relation rules will instead be denoted as $$\mathcal {C}_R$$. $$\mathcal {C}_R( a , b )$$ applies template $$\mathcal {C}_R$$ to $$ a , b \in { A}$$. $$ CoExistence ( a , b )$$ is a relation rule expressing that both $$ RespondedExistence ( a , b )$$ and $$ RespondedExistence ( b , a )$$ hold true: if $$ a $$ is carried out, also $$ b $$ must be carried out, and the other way around. $${ Precedence }( a , b )$$ is the relation rule establishing that, if $$ b $$ is carried out, then $$ a $$ must have been carried out *beforehand* at least once. $${ Precedence }( a , b )$$ not only imposes that to the execution of $$ b $$ corresponds an execution of $$ a $$—as $$ RespondedExistence ( b , a )$$—but it also requires that the execution of $$ b $$ be *preceded* by such execution of $$ a $$, i.e., it adds a condition over the *ordering* of the constrained activities. Therefore, $${ Precedence }( a , b )$$ falls under the category of *ordering relation* rules. Templates of such category will be denoted as $$\mathcal {C}_R^{\rightarrow }$$. Furthermore, by definition we have that if $${ Precedence }( a , b )$$ holds true, then $$ RespondedExistence ( b , a )$$ holds true as well. We thus say that $${ Precedence }( a , b )$$ *is subsumed by* $$ RespondedExistence ( b , a )$$. $$\mathcal {C}_R^{\rightarrow }( a , b )$$ indicates an *ordering relation* rule applied to $$ a , b \in { A}$$. In particular, $$\mathcal {C}_R^{\rightarrow }( a , b )$$ always specifies the order in which the occurrences of $$ a $$ and $$ b $$ are considered: $$ a $$ first, $$ b $$ afterward (henceforth, *order direction*).

In turn, $$ AlternatePrecedence ( a , b )$$ $${ Precedence }( a , b )$$ because (i) the former entails the latter, i.e., an execution of $$ a $$ must precede $$ b $$, and (ii) after the execution of $$ a $$ and of the subsequent $$ b $$, $$ b $$ cannot be carried out again, until $$ a $$ is performed again. The subsumption relation is transitive by definition. Therefore, $$ AlternatePrecedence ( a , b )$$ is also subsumed by $$ RespondedExistence ( b , a )$$.

Finally, $$ ChainPrecedence ( a , b )$$ is the last rule along the “Precedence” subsumption hierarchy as it is even more restrictive than $$ AlternatePrecedence ( a , b )$$: $$ a $$ must be executed before $$ b $$ and no other task can be carried out between $$ a $$’s and $$ b $$’s. $$ Succession ( a , b )$$ imposes that $$ a $$ must precede $$ b $$, just as $${ Precedence }( a , b )$$ does, but also the other way round: after $$ a $$, $$ b $$ must be carried out. $$ AlternateSuccession ( a , b )$$ is subsumed by $$ Succession ( a , b )$$. It restricts the condition exerted by the subsuming rule by stating that $$ a $$ and $$ b $$ must alternate to each other. In turn, $$ ChainSuccession ( a , b )$$ is subsumed by $$ AlternateSuccession ( a , b )$$ because it additionally imposes that no other task can be performed in between. $$ NotSuccession ( a , b )$$ specifies that once $$ a $$ is carried out, then no $$ b $$ can be performed after, and that $$ a $$ cannot precede $$ b $$. $$ NotCoExistence ( a , b )$$ is even stricter (and as such subsumed), because it imposes that $$ a $$ and $$ b $$ cannot both be performed in the context of the same process instance.

**Table 3:** Used Declare rules

Rule	Explanation	Cat.	Positive and negative examples			
$\textit{Participation}( a )$	$a$ occurs at least *once*	$\mathcal {C}_E( a )$	$\checkmark$ $bcac$	$\checkmark$ $bcaac$	$\times$ $bcc$	$\times$ $c$
$$ Init ( a )$$	$$ a $$ is the *first* to occur	$$\mathcal {C}_E( a )$$	$$\checkmark $$ $$ acc $$	$$\checkmark $$ $$ abac $$	$$\times $$ $$ cc $$	$$\times $$ $$ bac $$
$$ End ( a )$$	$$ a $$ is the *last* to occur	$$\mathcal {C}_E( a )$$	$$\checkmark $$ $$ bca $$	$$\checkmark $$ $$ baca $$	$$\times $$ $$ bc $$	$$\times $$ $$ bac $$
$RespondedExistence ( a , b )$	If $a$ occurs in the trace, then $b$ occurs as well	$\mathcal {C}_R( a , b )$	$\checkmark$ $bcaac$	$\checkmark$ $bcc$	$\times$ $caac$	$\times$ $acc$
$$ Precedence ( a , b )$$	$$ b $$ occurs only if preceded by $$ a $$	$$\mathcal {C}_R^{\rightarrow }( a , b )$$	$$\checkmark $$ $$ cacbb $$	$$\checkmark $$ $$ acc $$	$$\times $$ $$ ccbb $$	$$\times $$ $$ bacc $$
$$ AlternatePrecedence ( a , b )$$	Each time $$ b $$ occurs, it is preceded by $$ a $$ and no other $$ b $$ can recur in between	$$\mathcal {C}_R^{\rightarrow }( a , b )$$	$$\checkmark $$ $$ cacba $$	$$\checkmark $$ $$ abcaacb $$	$$\times $$ $$ cacbba $$	$$\times $$ $$ acbb $$
$$ ChainPrecedence ( a , b )$$	Each time $$ b $$ occurs, then $$ a $$ occurs immediately beforehand	$$\mathcal {C}_R^{\rightarrow }( a , b )$$	$$\checkmark $$ $$ abca $$	$$\checkmark $$ $$ abaabc $$	$$\times $$ $$ bca $$	$$\times $$ $$ bacb $$
$$ CoExistence ( a , b )$$	If $$ b $$ occurs, then $$ a $$ occurs, and vice versa	$$\mathcal {C}_R( a , b )$$	$$\checkmark $$ $$ cacbb $$	$$\checkmark $$ $$ bcca $$	$$\times $$ $$ cac $$	$$\times $$ $$ bcc $$
$$ Succession ( a , b )$$	$$ a $$ occurs if and only if it is followed by $$ b $$	$$\mathcal {C}_R^{\rightarrow }( a , b )$$	$$\checkmark $$ $$ cacbb $$	$$\checkmark $$ $$ accb $$	$$\times $$ $$ bac $$	$$\times $$ $$ bcca $$
$$ AlternateSuccession ( a , b )$$	$$ a $$ and $$ b $$ if and only if the latter follows the former, and they alternate each other in the trace	$$\mathcal {C}_R^{\rightarrow }( a , b )$$	$$\checkmark $$ $$ cacbab $$	$$\checkmark $$ $$ abcabc $$	$$\times $$ $$ caacbb $$	$$\times $$ $$ bac $$
$$ ChainSuccession ( a , b )$$	$$ a $$ and $$ b $$ occur if and only if the latter immediately follows the former	$$\mathcal {C}_R^{\rightarrow }( a , b )$$	$$\checkmark $$ $$ cabab $$	$$\checkmark $$ $$ ccc $$	$$\times $$ $$ cacb $$	$$\times $$ $$ cbac $$
$$ NotSuccession ( a , b )$$	$$ a $$ can never occur before $$ b $$	$$\mathcal {C}_R^{\rightarrow }( b , a )$$	$$\checkmark $$ $$ bbcaa $$	$$\checkmark $$ $$ cbbca $$	$$\times $$ $$ aacbb $$	$$\times $$ $$ abb $$
$$ NotCoExistence ( a , b )$$	$$ a $$ and $$ b $$ never occur together	$$\mathcal {C}_R( a , b )$$	$$\checkmark $$ $$ cccbbb $$	$$\checkmark $$ $$ ccac $$	$$\times $$ $$ accbb $$	$$\times $$ $$ bcac $$

The concept of subsumption also applies to the existence rules. For instance, both $$ Init ( a )$$ and $$ End ( a )$$ are existence rules subsumed by $$ Participation ( a )$$, because (i) they both impose that $$ a $$ must be carried out in every process instance, as per $$ Participation ( a )$$, and (ii) they, respectively, establish that $$ a $$ must be the first ($$ Init $$) or the last ($$ End $$) activity performed [16].

We remark here that Declare rule templates are not independent of one another. Indeed, subsumed constraints always entail the subsuming ones, as e.g., in the aforementioned cases of $$ Init ( a )$$ and $$ Participation ( a )$$ or $$ ChainSuccession ( a , b )$$ and $$ AlternateSuccession ( a , b )$$. Furthermore, constraints such as $$ Succession ( a , b )$$ entail by definition $${ Precedence }( a , b )$$. Without loss of generality, we will thus consider in the following explanatory examples the strictest constraints. A subset of the subsumed and entailed constraints will be optionally mentioned for the sake of clarity.

Taking inspiration from the tabular representation of behavioral relations in [47, 48], we formally define a Declare model $M_{\textsf {D}}$ as a tuple $M_{\textsf {D}}= \left\langle { A}, \mathcal {C}_E, \mathcal {C}_R, \varepsilon _E, \varepsilon _R, {\mathbb {B}} \right\rangle$, where: *A* is the set of activities; $\mathcal {C}_E$ is the repertoire of existence rule templates; $\mathcal {C}_R$ is the repertoire of relation rule templates (we recall here that ordering relation rule templates constitute a strict subset of it, $\mathcal {C}_R^{\rightarrow }\subset \mathcal {C}_R$); ${\mathbb {B}}$ is the set of boolean values $\textit{true}$ and $\textit{false}$; $\varepsilon _E: \mathcal {C}_E\times { A}\rightarrow {\mathbb {B}}$ is the evaluation function over existence rules, specifying whether an existence rule template holds true, applied to an activity; $\varepsilon _R: \mathcal {C}_R\times { A}\times { A}\rightarrow {\mathbb {B}}$ is the evaluation function over relation rules specifying whether a relation rule template holds true applied to a pair of activities.

As said, events are meant to be recordings of the activities carried out during the process enactment. Therefore, we will interchangeably interpret Declare rules as (i) behavioral relations between activities in a process model or (ii) conditions exerted on the occurrence of events in traces. The latter is typical in the context of Declare mining [21, 40]. Notice that it is a different approach than the former, typically used for Declare modeling as originally conceived by the seminal work of Pesic [45]. With a slight abuse of notation, we will henceforth also consider, e.g., $$ NotCoExistence (o,p)$$ with $$o,p \in E $$ to specify that *events* *o* and *p* cannot occur in the same *trace*.

Table 3 lists the set of Declare rules that form the base of the behavioral matching presented in the remainder of the paper. Each Declare rule is assigned to one of the previously defined *categories* (i.e., either $\mathcal {C}_E$, $\mathcal {C}_R$ or $\mathcal {C}_R^{\rightarrow }$). For every rule, two examples of complying traces and two examples of violating traces are provided. The complete list of Declare rule templates can be found in [21, 53].

In light of the above, we can analyze some constraints that are satisfied in the log of Fig. 1. The existence constraints $$ Participation ( Person )$$, $$ Participation ( Details )$$, and $$ Participation ( Status )$$ are satisfied, because such events occur in every trace. $$ End ( Status )$$ is satisfied too, because every trace not only contains a $$ Status $$ event, but also *terminates* with that event. Considering the relation rules, e.g., $$ RespondedExistence ( Protocol , CI )$$ is satisfied. Please notice that this does not hold true for $$ RespondedExistence ( CI , Protocol )$$, because $$ CI $$ occurs in the traces of Case 2 and Case 5, whereas no $$ Protocol $$ is in them. However, a stricter constraint can be indicated as valid, namely $${ Precedence }( CI , Protocol )$$, because all $$ Protocol $$ events are preceded by $$ CI $$—in the traces where they occur. We can proceed deeper in the subsumption hierarchy and state that $$ AlternatePrecedence ( CI , Protocol )$$ is satisfied, because no other $$ Protocol $$ event occurs in between. In contrast, although $${ Precedence }( Details , CI )$$ is satisfied, $$ AlternatePrecedence ( Details , CI )$$ is violated in traces 3 and 5. $$ ChainPrecedence ( CI , Group )$$ is also valid in the log, as well as $$ ChainPrecedence ( Group , Comment )$$. Moreover, $$ ChainSuccession ( Comment , Protocol )$$ is verified, because the two events always occur in the same order and one after the other. On the contrary, $$ ChainSuccession ( CI , Group )$$ is not verified, because in traces 2 and 5 there is no $$ Group $$ right after $$ CI $$, and in trace 3 $$ Group $$ is repeated before $$ Group $$. $$ AlternateSuccession ( Person , Classification )$$ is valid in the log, because the latter event always occurs after the former, without any recurrence of $$ Person $$ or $$ Classification $$ in between. This is not true for $$ Details $$ and $$ Classification $$, because $$ Details $$ recurs in between in traces 1 and 3—as a consequence, $$ AlternateSuccession ( Details , Classification )$$ cannot be indicated as valid in the log. $$ AlternateSuccession ( Person , Classification )$$ and $$ AlternateSuccession ( Person , Status )$$ are valid instead.

Declare rules that are discovered from event logs are usually associated to a reliability metric, namely *support* [21, 40]. Support is a normalized value ranging from 0 to 1 that measures to what extent traces are compliant with a rule. A support of 0 stands for a rule which is always violated. Conversely, a value of 1 is assigned to the support of rules which always hold true. According to the measurement introduced by the work of [21], the analysis of a trace $$t_1 = \left[ b , a , c , b , a , b , b , c \right] $$ would lead to a support of 1 to $$ Participation ( a )$$, 0 to $$ NotCoExistence ( a , b )$$, and 0.75 to $${ Precedence }( a , b )$$, as 3 $$ b $$’s out of 4 are preceded by an occurrence of $$ a $$. Considering an event log, which consists of $$t_1$$ and $$t_2 = \left[ c , c , a , c , b \right] $$, the support of $$ Participation ( a )$$ and $$ NotCoExistence ( a , b )$$ would remain equal to 1 and 0, respectively, whereas the support of $${ Precedence }( a , b )$$ would be 0.8 (4 $$ b $$’s out of 5 are preceded by an occurrence of $$ a $$). [21] provides further details on the computation of support values for each rule. Some rules that are not fully supported in the log of Fig. 1, e.g., are: (i) $$ Init ( Person )$$, having a support of $$0.8\bar{3}$$, because only 5 traces out of 6 start with that event; (ii) $$ ChainPrecedence ( Details , Classification )$$, having a support of $$0.8\bar{3}$$ too, because only 5 $$ Classification $$ events out of 6 are directly preceded by $$ Details $$; (iii) $$ AlternatePrecedence ( Classification , CI )$$, having a support of 0.5, because only 3 $$ CI $$ events out of 6 are preceded by $$ Classification $$ without other $$ CI $$’s in between; (iv) $${ Precedence }( Protocol , Status )$$, having a support of $$0.\bar{3}$$, because only 2 $$ Status $$ events out of 6 are preceded by $$ Protocol $$.

Such a metric is usually utilized to prune out those rules that are associated to a value below a user-defined threshold. The rationale behind the choice of the support is the balance between (i) the non-frequent behavior that the user does not want included in the discovered model, and (ii) the amount of noise that is supposed to affect the log. Indeed, higher thresholds cause the discovered model to retain only those rules that define the most frequent behavior. Therefore, less violations to the rules are permitted in the log. Such violations could be due to noise in the log though, in terms of incorrectly recorded events. Referring to the example of Fig. 1, it could be that an incorrect registration of “Person added” and “Details logged” events caused the inverse order of trace 6. However, such a recording error would make the $$ Init ( Person )$$ and $$ ChainPrecedence ( Details , Classification )$$ rules be discarded anyway with a threshold of $$85\%$$.

### Modeling of activity life cycles

**Fig. 2:**
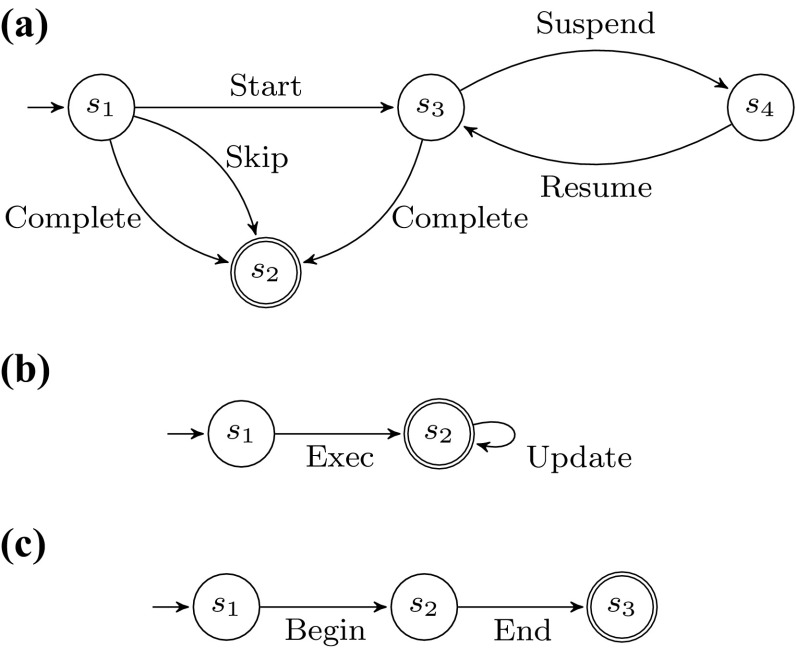
Examples of activity life-cycle models, depicted as finite state automata. **a** Model comprising either a skipping of the activity, or start and completion, with optional intermediate alternations of suspensions and resumptions, **b** Model depicting the activity execution with further repeatable refinements, **c** Model with beginning and concluding transitions in sequence

When a process is executed, the activities of the corresponding process model are instantiated. In this paper, we consider that activities are not atomic: during the lifetime of an activity instance, the activity instance traverses different states. There are different life-cycle models proposed in the literature (e.g., [52, p. 101], [58, p. 83ff.]). In this paper, we adopt a simplified version of the life-cycle model proposed by van der Aalst in [52, p. 101]. There, the activity life cycle is modeled as a stateful artifact, evolving from an initial state to a final state by means of so-called *life-cycle transitions*. To this extent, the finite state automaton is the proposed formal model. Let $LCS$ be a set of states and $LT$ be the set of activity life-cycle transition labels. An activity life-cycle model $ALM= \left\langle LCS, lcs_I, LCS_F, LT, \theta \right\rangle$ is a finite state automaton that defines the allowed sequences of life-cycle transitions. $\theta \subseteq LCS\times LT\times LCS$ is the (labeled) transition relation modeling the allowed life-cycle transitions in a given state. An activity life-cycle model has an initial state $lcs_I \in LCS$ and final states $LCS_F \subseteq LCS$. Different activities in the process can be associated to different life-cycle models. Figure 2 shows three examples of activity life-cycle models. The model of Fig. 2a, $ALM_{2\mathrm{(a)}}$, has $\left\{ s_1, \ldots , s_4 \right\}$ as the states set, $s_1$ as the initial state, singleton $\left\{ s_2 \right\}$ as the final states set, $\left\{ \text {Start}, \text {Skip}, \text {Suspend}, \text {Resume}, \text {Complete}\right\}$ as the activity life-cycle transitions, and the following transition relation: $\{ \left\langle s_1, \text {Start}, s_3 \right\rangle$, $\left\langle s_1, \text {Complete}, s_2 \right\rangle$, $\left\langle s_1, \text {Skip}, s_2 \right\rangle$, $\left\langle s_3, \text {Suspend}, s_4 \right\rangle$, $\left\langle s_4, \text {Resume}, s_3 \right\rangle , \left\langle s_3, \text {Complete}, s_2 \right\rangle \}$. Likewise, in Fig. 2b the depicted model corresponds to automaton $ALM_{2\mathrm{(b)}}=\langle \{ s_1, s_2 \}, s_1, \{s_2\}$, $\{\text {Exec},\text {Update}\}$, $\{ \left\langle s_1, \text {Exec}, s_2 \right\rangle$, $\left\langle s_2, \text {Update}, s_2 \right\rangle \} \rangle$. The automaton illustrated in Fig. 2c is $ALM_{2\mathrm{(c)}}=\langle \{ s_1, s_2, s_3 \}, s_1, \{s_3\}, \{\text {Begin},\text {End}\}$, $\{ \left\langle s_1, \text {Begin}, s_2 \right\rangle ,\left\langle s_2, \text {End}, s_3 \right\rangle \} \rangle$.

In the following, we assume that event classes in the event log reflect the enacted transition in the activity life-cycle model, i.e., an occurring event corresponds to a move in the activity life-cycle model dictated by its class. Considering, e.g., the example of Fig. 1 and assigning $ALM_{2\mathrm{(c)}}$ as the life-cycle model of activity “Functional escalation”, then event “Group” can represent transition $\left\langle s_1, \text {Begin}, s_2 \right\rangle$, and event “Comment” can correspond to transition $\left\langle s_2, \text {End}, s_3 \right\rangle$.

**Fig. 3:**
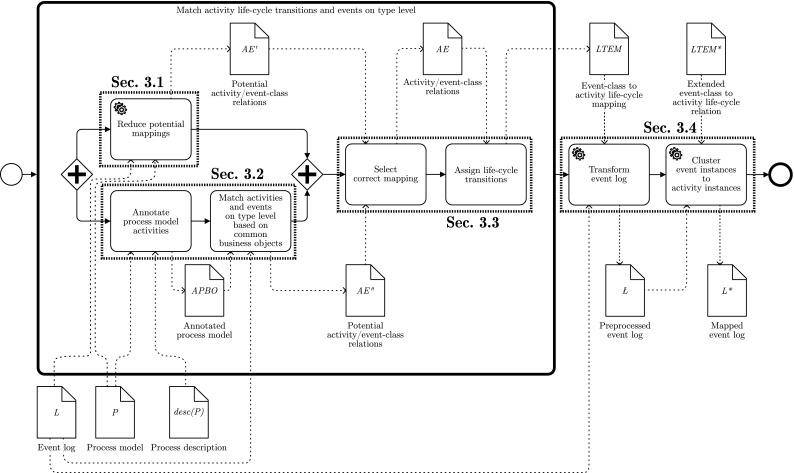
Overview of the matching approach

## Integrated matching approach

This section introduces our approach for the mapping of events to predefined activities of a process model. Figure 3 shows an overview of the workflow of the proposed solution with a BPMN-like notation. The three main steps of the approach are emphasized by bold boxes. First, a *mapping on type level* is established between events and activities. Second, the type-level mapping is used to *transform the event log* in such a way that each event instance is related to its corresponding activity life-cycle transition. Finally, the event instances are clustered into activity instances.

During the first step (the matching on the type level), two different perspectives are taken into account in order to find correspondences between event classes and model activities: the behavioral and the label perspectives. The adopted techniques are, respectively, detailed in Sects. 3.1 and 3.2. They are executed in parallel, as represented by the AND gateways at the sides. Here, we reuse and extend previous work from [4, 5]. For each perspective, a set of potential event-activity relations is derived ($$AE', AE'' \subseteq { A}\times E$$). Both relations are used in a subsequent filtering step to achieve the correct mapping using a questionnaire-driven user interaction. The outcome ($$AE\subseteq { A}\times E$$) associates every event class to an activity. With such a mapping, the help of the analyst is requested to annotate to which event classes the life-cycle transitions can be assigned with respect to the related activity. Only life-cycle transitions other than “Start” and “Complete” need to be linked. “Start” and “Complete” transitions will be discovered automatically in the last step. The annotation of life-cycle transitions leads to the mapping relation $$ LTEM \subseteq LT\times { A}\times E$$, which is then used for the first transformation of the event log. During the transformation, each event instance is relabeled according to the mapping provided in $$ LTEM $$. As we do not require a complete mapping of event classes to life-cycle transitions, the preprocessed event log is not yet aware of activity instances. That is, it is not clear when a new activity instance actually starts and ends. Therefore, the last step employs a clustering technique that takes the so-called activity instance border definitions as an input from the user ($$ LTEM ^*$$). These activity instance borders define how to identify the existence of multiple activity instances. After the clustering, the final mapped event log $$L^*$$ is returned and can be used with any of the available process mining techniques. The following sections provide the details for each of the steps.

### Type-level matching using Declare rules

This section describes how the automated step “Reduce potential mappings” from Fig. 3 is implemented in order to derive the first set of potential activity event class relations ($AE'$). To this end, a constraint satisfaction problem (CSP) is defined to restrict the possible mappings of events and activities. A CSP is a triple $CSP = \left\langle X , D, C \right\rangle$ where $X = \langle x_1, x_2, \dots , x_v \rangle$ is a *v*-tuple of variables with the corresponding domains specified in the *v*-tuple $D = \langle D_1, D_2, \dots , D_v \rangle$ such that $x_i \in D_i$ [28]. $C = \langle c_1, c_2, \dots , c_t \rangle$ is a *t*-tuple of constraints. We use predicate logic to express the constraints used in this paper. The set of solutions to a CSP is denoted as $S = \{ S _1, S _2, \dots , S _r \}$ where each solution $S _k = \langle s _1, s _2, \ldots , s _v \rangle$ is a *v*-tuple with $k \in 1..r$, $s _i \in D_i$ and such that every constraint in *C* is satisfied.

To build the CSP, the activities and event labels need to be mapped to the set of variables and their domains. Therefore, a bijective function ${ var : E \rightarrow X}$ is defined that assigns each event label to a variable with the natural numbers $1..|{ A}|$ as domain. Furthermore, a bijective function $val : { A}\rightarrow 1..|{ A}|$ is defined that assigns each activity a natural number in the range from 1 to the number of activities. Table 4a, b shows the mapping $var$ and the mapping $val$ for our example.

**Table 4:** Mapping of activities and event labels

(a) Mapping $var$	
Variable $var (e) \in X$	Event $e \in E$
$$ x _1$$	CI selected
$$ x _2$$	Classification specified
$$ x _3$$	New comment created
$$ x _4$$	Details logged
$$ x _5$$	Group changed
$$ x _6$$	KB update performed
$$ x _7$$	Person added
$$ x _8$$	New protocol created
$$ x _9$$	Solution assigned
$$ x _{10}$$	Status changed

(b) Mapping $val$	
Value $val(a) \in 1..|{ A}|$	Activity $a\in { A}$
1	Incident logging
2	Incident classification
3	Initial diagnosis
4	Functional escalation
5	Investigation and diagnosis
6	Resolution and recovery
7	Incident closure

With the variables and domains defined, the solutions to the CSP reflect all possible mappings between events and activities. For *n* activities and *m* events, there are potentially $n^m$ solutions. For example, these are $7^{10} = 282,475,249$ possible mappings. Yet, this also includes solutions where not all activities are assigned to an event or solutions where all events are mapped to one single activity. As these solutions are not desired, we first restrict the set of solutions to those that assign each activity to at least one event. Note that we assume that the execution of each activity in the process model is being logged by the supporting IT system. Thus, those activities that are not recorded are not considered in the processing. We assume that each event in the given log relates to exactly one activity in the process model, whereas one activity can relate to multiple events. Thus, we are using the NVALUE constraint available in many constraint problem solvers [28]. This constraint ensures that each value in the domain of the variables is assigned at least once. Still, the complexity of the matching problem remains very high. In the following, we present an approach to tackle this complexity issue by combining the information available in the log with knowledge on the process model structure.

#### Discovery of Declare rules

In order to reduce the number of possible mappings between activities and events on type level, we look at Declare rules describing the behavior of event logs and process models.

To derive such rules from the event logs, we utilize the techniques explained in [21]. The approach of [21], named MINERful, is among the fastest automated discovery algorithms for declarative processes, and is based upon a two-phase computation. The first one creates a so-called knowledge base. It contains the statistics about the occurrences and positions of events. For our examples, we again use a log consisting of traces $t_1 = \left[ b , a , c , b , a , b , b , c \right]$ and $t_2 = \left[ c , c , a , c , b \right]$. For each event class, e.g., $a$ and $b$, the registered information pertains the occurrences and positions of the related events. This information relates to: (i) Events taken singularly—e.g., the number of traces in which $a$ occurred at least once in the log (2 in the example), or the number of times in which $b$ occurred as the first event in the trace (1 in the example), and (ii) Event pairs in relation to one another—e.g., the number of $b$ events that occurred without being preceded by an event of class $a$ in the same trace (1 in the example).

The second phase is dedicated to the computing of the rules’ support by querying the knowledge base. In particular, arithmetical operations on gathered information are performed to obtain a value ranging from 0.0 to 1.0 that represents the frequency with which rules are satisfied in the log. For example, the support of $$ Participation ( a )$$ amounts to the number of traces where $$ a $$ occurs, divided by the number of traces in the log (hence, 2 / 2 in the example, namely 1.0). The support of $$ Init ( b )$$ corresponds to the number of times in which $$ b $$ occurs as the first event of the traces, again scaled by the number of traces in the log (1 / 2 in the example, hence 0.5). The support of a relation constraint such as $${ Precedence }( a , b )$$ proceeds as follows: First, the number of $$ b $$ events occurring without a preceding $$ a $$ is scaled by the number of $$ b $$’s occurring in the log (1 / 5, hence 0.2 in the example); thereafter, such quantity is subtracted to 1.0 (the support of $${ Precedence }( a , b )$$ is thus equal to 0.8). The complete explanation of how MINERful works and the description of the theory behind it can be found in [21].

We refer to a *simulation log* as a generated synthetic event log such that at least one trace is recorded for each legal path in the process model. In order to infer Declare rules from process models, we build upon the following assumption: The Declare rules that are satisfied with a support of 100% in the simulation log reflect the behavior of the original process model [13, 22]. Therefore, we derive the corresponding Declare constraints as follows. We generate a synthetic event log using the simulation technique described in [49]. As said, the simulation log is built so as to contain every execution path represented as a trace.^3^ Thereafter, we apply the discovery algorithm of [21] on it to derive the Declare rules that have a support of 100%. Because all traces of the simulation log comply with such rules, and those traces represent all the possible executions of the model by construction, the rules inferred from the simulation log are those ones that hold true in the model. Rules that are not compliant with the model would not have a support of 100% because there would be at least a trace in which they do not hold true. From the process model of the example depicted in Fig. 1, $$ Init ( Incident~logging )$$ is inferred, as well as $$ End ( Incident~closure )$$. $$ AlternateSuccession ( Incident~logging , Incident~closure )$$ is also part of the declarative rules that derive from the model, because “Incident logging” and “Incident closure” are, respectively, the first and the last activity to be performed, they are not involved in any loops, and other tasks need to be carried out in between. Furthermore, the $$ Participation $$ rule holds true for all activities but “Functional escalation” and “Investigation and diagnosis”, which in fact lie on an alternative branch following an XOR gateway and are thus optional.

We denote the set of Declare rules inferred from the process model and its simulation log as $$\mathcal {B}_{M}$$, to distinguish them from the set of all Declare rules discovered from the original event log, namely $$\mathcal {B}_{L}$$. We classified the introduced Declare rules into three different categories, namely existence rules $$\mathcal {C}_E$$, relation rules $$\mathcal {C}_R$$ and ordering rules $$\mathcal {C}_R^{\rightarrow }$$. We make use of this categorization by handling all rules that are classified as ordering rules ($$\mathcal {C}_R^{\rightarrow }$$) as a single rule, giving an ordering between elements (i.e., either between activities or between events). Therefore, only the ordering rule with the highest support is kept for each pair of elements. In the example event log of Fig. 1, e.g., $$ AlternatePrecedence ( Person , CI )$$ and $${ Precedence }( Person , CI )$$ have a support of $$66.\bar{6}\%$$ and $$100.0\%$$, respectively. Therefore, only $${ Precedence }( Person , CI )$$ is retained. As an ordering rule may entail other ordering rules, there may be multiple ordering rules for a pair of elements, of which all rules obtain the highest support. In such a case, we retain among those the ones which are not entailed by the other rules, following the approach of [17, 39]. In the following, we use $$\mathcal {C}_R^{\rightarrow }(e_1, e_2)$$ to refer to the chosen ordering relation for a pair of event classes $$(e_1, e_2)$$ with the highest support. Similarly, $$\mathcal {C}_R^{\rightarrow }(a_1, a_2)$$ is used to denote a rule on a pair of activities.

Beyond the defined Declare rules, a set of interleaving elements $\mathcal {I}\subseteq ({ A}\times { A}) \cup (E\times E)$ is introduced. In case there is no ordering rule with a support above $\beta$ for a given pair of elements, we add the pair to the set of interleaving elements.

#### Building of the constraint satisfaction problem

Having the Declare rules from both the model and the event log as well as the set of interleaving pairs of events/activities, we can define constraints to reduce the number of possible mappings between event classes and activities. To define the constraints described here, we also took inspiration from a previous study in the literature by Leopold et al. [37], who devised a collection of behavioral relations for the semantic matching of process models.

Starting with the ordering rules, formula (1) provides the corresponding constraint for rules in $\mathcal {C}_R^{\rightarrow }$. If two event classes are in an ordering relation and mapped to two different activities, these activities also have to be in an ordering relation enforcing the same order direction.

Note that in formula (1) as well as in all upcoming formulas $e_1$, $e_2 \in E$ denote two different event classes, i.e.,  $e_1 \ne e_2$. In the same manner, $a_1$, $a_2 \in { A}$ denote two different activities, i.e.,  $a_1 \ne a_2$.

1 $$\begin{aligned} \begin{aligned}&\mathcal {C}_R^{\rightarrow }(e_1, e_2) \wedge Map\left( e_1\right) =a_1 \wedge Map\left( e_2\right) =a_2 \\&\quad \implies \mathcal {C}_R^{\rightarrow }(a_1, a_2) \end{aligned} \end{aligned}$$

In the example of Fig. 1, e.g., an ordering relation holds between $$ Person $$ events and the following $$ Status $$ ones (cf. $$ AlternateSuccession ( Person , Status )$$, as seen in Sect. 2.3). A mapping that associates $$ Person $$ to “Incident logging” and $$ Status $$ to “Incident closure” satisfies the related constraint 1, because it is also true that an ordering relation rule holds between “Incident logging” and “Incident closure” (cf. $$ AlternateSuccession ( Incident~logging , Incident~closure )$$, as seen in Sect. 3.1.1). By the same line of reasoning, also a mapping that associates $$ Person $$ and $$ Status $$ to “Incident logging” and “Incident classification” would be correct, considering this constraint alone. The mapping of $$ Person $$ to “Initial diagnosis” and of $$ Status $$ to “Incident logging” has to be excluded instead because it would violate the constraint: It is indeed false that “Initial diagnosis” has to be executed before “Incident logging” in the process model.

Formula (2) adds the constraint for pairs of event classes that are exclusive to each other and thus result in a rule of the type NotCoExistence. Again, such a pair of event classes can only be mapped to a pair of exclusive activities or to the same activity.

2 $$\begin{aligned} \begin{aligned}&NotCoExistence (e_1,e_2) \; \wedge Map\left( e_1\right) =a_1 \\&\quad \wedge Map\left( e_2\right) =a_2 \implies NotCoExistence (a_1,a_2) \end{aligned} \end{aligned}$$

Regarding the pairs of events that are not exclusive and for which no ordering rule exceeds the minimum support $\beta$, formula (3) ensures that if a pair of interleaving events is mapped to a pair of activities, these activities are also in interleaving order.

3 $$\begin{aligned} \begin{aligned}&(e_1, e_2) \in \mathcal {I}\; \wedge Map\left( e_1\right) =a_1 \wedge Map\left( e_2\right) =a_2 \\&\quad \implies (a_1, a_2) \in \mathcal {I}\end{aligned} \end{aligned}$$

The category of relation rules ($\mathcal {C}_R$) furthermore includes the $CoExistence$ rule. If two event classes that are co-existing are matched to two different activities, these activities should also be co-existing, as defined in formula (4).

4 $$\begin{aligned} \begin{aligned} CoExistence (e_1,e_2)&\wedge Map\left( e_1\right) =a_1 \\&\wedge Map\left( e_2\right) =a_2 \\&\implies CoExistence (a_1,a_2) \end{aligned} \end{aligned}$$

Besides the already used Declare rules, there are further Declare rules that can be leveraged to build constraints reducing the number of possible solutions. That is, the Declare approach also makes use of the rules classified as existence rules ($\mathcal {C}_E$). The constraint introduced in formula (5) ensures that events for which an $Init$ rule exists are only mapped to activities for which an $Init$ rule exists. Formulas (6) and (7) work in the same manner for $End$ and $Participation$ rules.

5 $$\begin{aligned} Init (e_1)&\;\wedge \, Map\left( e_1\right) =a_1 \implies Init (a_1) \end{aligned}$$

6 $$\begin{aligned} End (e_1)&\;\wedge \, Map\left( e_1\right) =a_1 \implies End (a_1) \end{aligned}$$

7 $$\begin{aligned} Participation (e_1)&\;\wedge \, Map\left( e_1\right) =a_1\nonumber \\&\implies Participation (a_1) \end{aligned}$$

Considering the example of Fig. 1, constraint 6 guarantees that “Status changed” can be only mapped to “Incident closure”, because they are the only event class and activity for which $End$ hold true (see Sects. 2.3, 3.1.1). Because “Person added”, “Details logged”, and “Status changed” are all subject to the $Participation$ rule in the log, then constraint 7 avoids that they are mapped to “Functional escalation” or “Investigation and diagnosis”.

Having the constraint definitions in the propositional formulas 1–7, a constraint ${c_i, i \in 1..|\mathcal {B}_{L}|}$ is added to the CSP for each Declare rule derived from the event log as per Sect. 3.1.1. Note that a certain degree of noise is handled already by accepting behavioral relations and declarative rules with a support less than 1.0.

#### Constraints for special cases

In the course of our preliminary experiments with synthetic and real-life event logs, bringing about the studies reported in this paper, we have noticed that the constraints defined in the previous section may be too strict in some cases due to the fact that not all behavior of a process is observed equally often. To this end, mandatory events as well as *interleaving* and *co-occurrence relations* can play a special role.

First of all, *mandatory* events may under certain circumstances also belong to optional activities. Consider the case where the event “New protocol created”, which belongs to the optional activity “Investigation and diagnosis”, is seen in more than 90% of the traces of the event log. If the minimum threshold $\beta$ set is lower than or equal to the relative observations of “New protocol created” events, a $Participation$ rule is discovered for “New protocol created” and formula (7) leads to the exclusion of the correct mapping. We recall here that the choice of $\beta$ below 1.0 determines the balance between the amount of non-frequent behavior to include in the whole analysis and the amount of noise to exclude from it. Therefore, in order to avoid that lower values for the threshold lead to incorrect mappings in such cases, we define formula (7) as an optional constraint that can be omitted.

The same phenomenon also influences constraints stemming from *co-occurrence relations* that suffer from the fact that some behavior is seen more often than other. If an event stemming from an optional activity generates a $Participation$ rule, this also leads to the derivation of co-occurrence relations with all events that also occur more often than the defined threshold. That is, there is for example a co-occurrence relation for “New protocol created” and “Details given”, which belongs to the mandatory activity “Incident logging”. Yet, in the model the two activities “Incident logging” and “Investigation and diagnosis” are not in a co-occurrence relation since the latter activity is optional. Hence, cases where optional activities are executed almost always lead to problems with co-occurrence constraints as they disallow the correct mapping. In order to tackle this problem, a relaxed constraint definition for co-occurrence constraints is introduced in formula (8).

8 $$\begin{aligned} \begin{aligned}&CoExistence (e_1,e_2) \wedge Map\left( e_1\right) =a_1 \wedge Map\left( e_2\right) =a_2 \\&\quad \implies \lnot NotCoExistence (a_1,a_2) \end{aligned} \end{aligned}$$

The *relaxed co-occurrence* constraints defined in formula (8) forbid two events that are found to be in a co-occurrence relation to be mapped to two activities that are exclusive to each other. Thereby, the basic co-occurrence constraint is relaxed as we do not require the two matching activities to be in a co-occurrence relation. This allows us to handle cases where optional activities are executed very frequently, while still making use of the co-occurrence relations for the pruning of unwanted mappings.

Additionally, *interleaving relations* might not always be reflected in the execution. To give an example, consider a small change in the incident process example that makes the activities “Incident classification” and “Initial diagnosis” concurrent. Yet, the corresponding event classes “Classification specified” and “CI selected” are in an ordering relation, because “Classification specified” always occurs directly before “CI selected”. Such a situation is still coherent with respect to the model. Therefore, formula (9) introduces a different handling of event classes that are in an ordering relation.

9 $$\begin{aligned} \begin{aligned}&\mathcal {C}_R^{\rightarrow }(e_1, e_2) \wedge Map\left( e_1\right) =a_1 \wedge Map\left( e_2\right) =a_2 \\&\quad \implies \mathcal {C}_R^{\rightarrow }(a_1, a_2) \vee (a_1, a_2) \in \mathcal {I}\end{aligned} \end{aligned}$$

If two event classes in an order relation are mapped to two different activities, these activities have to be either also in an ordering relation or in interleaving order. As the newly introduced constraint allows for more matchings with respect to the ordering relations in the process model than its base counterpart formula (1), it is called *relaxed ordering* constraint. We specifically introduce this as a different notion to give the analyst the choice to use the relaxed ordering constraint or the (basic) ordering constraint. The reason for this choice is that the relaxed ordering constraint may introduce quite a number of potential matches that are not wanted, because every pair of ordered event classes that actually maps to a pair of activities in ordering relation can now also map to all pairs of interleaving activities. If it is known that events belonging to interleaving activities are also seen in all possible orderings equally often, one should not use the relaxed ordering constraint, but rather the constraint defined in formula (1).

Finally, we observed in our validation and evaluation with synthetic and real-life event logs that the interleaving constraints are especially sensitive toward noise. The noise sensitivity of interleaving constraints is due to the fact that each ordering relation turns into an interleaving relation when it is violated too often to be seen as an ordering relation. Therefore, we make the interleaving constraints optional and let the analyst decide whether to use them or not. The interleaving constraints should only be left out if a log is known to be noisy, as the exclusion of constraints typically increases the number of potential solutions.

### Type-level matching using label analysis

**Fig. 4:**
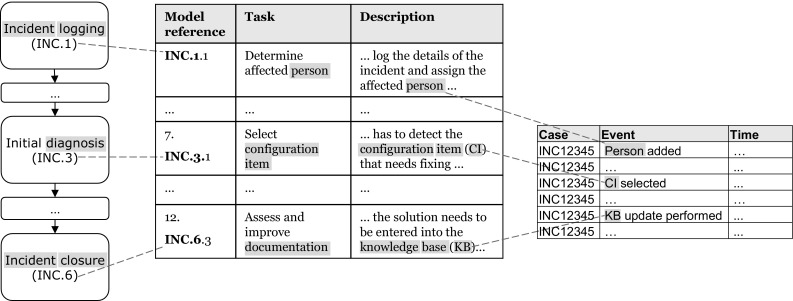
Connection of events to activities based on the description and the work instructions

Coming from the behavioral analysis for matching activities and events on type level, we now turn to a different perspective: the activity and event labels. In order to utilize the labels of events and activities, we employ the label analysis technique introduced in [5]. The technique is composed of two steps. First, the model activities are annotated with textual descriptions. These annotations serve the purpose of enriching the coarse-granular activities of the process model with detailed information that helps to link to events. In modern business process modeling tools, activities can be connected with more detailed textual descriptions, such that the annotation of the activities is readily available. Often, instructions can also be found in tabular form consisting of columns for the activity name and the detailed description, as in our incident process example in Table 1. In the following, we assume that such a description is available or can be directly linked to an activity.

In order to effectively use the activity descriptions for the matching of event classes and activity types, we have to preprocess the descriptions. As events often represent some kind of change to an object, we are especially interested in the objects contained in the activity descriptions. Therefore, the Stanford Part-of-Speech (POS) tagger [33, 51] is used to filter out these objects. The POS tagger parses natural text and assigns each word to its part of speech, e.g., verb, noun, article, adjective. From these categories, we only take into account words that are nouns or words for which no real category can be found by the POS tagger. The latter are most often abbreviations, such as “CI” or foreign words. Furthermore, all numbers are filtered out. The goal is to extract potential business objects. The set of all potential business objects is denoted as $PBO$. $PBO_{a} \subset PBO$ is the set of potential business objects $pbo_i \in PBO_{a}$ that unites all potential business objects for an activity $a\in { A}$. These objects are extracted from all activity description $ad\in desc(a)$, where $desc$ is a function mapping an activity to a set of textual descriptions, as seen in Table 1. Additionally, the labels of the activities are processed in the same way to extract further potential business objects. The activities are annotated with the derived objects for further processing in the next phase of the approach. The result of this phase is an activity annotation relation $APBO\subseteq { A}\times PBO$.

This relation is a many-to-many relations since one activity can be linked to multiple potential business objects and one potential business object can be associated with multiple different activities. Note that the annotation is not mandatory for each activity. Yet, it presumably improves the automated matching result because the textual descriptions are likely to be closer to the abstraction level of the event log than the activities in the process model as shown in [5].

Having annotated the activities with their potential business objects, the next step deals with the derivation of the activity-to-event-classes relation $AE$. To this end, we inspect each combination of event class and activity name as well as each combination of event class and activity description for potential correspondences.

In order to check for potential correspondences, we also derive the objects from the event classes in the same manner, yielding the relation $EPBO\subseteq E\times PBO$. Each tuple in $APBO$ is compared to each tuple in $EPBO$ by comparing the business objects.

As we aim for a high recall, we do not only make simple string comparisons in order to check the relatedness of two business objects. Rather, we employ natural language processing techniques as we explain in the following. Since we evaluate our approach with process models and logs written in German, we present examples that refer to this language and stem from our direct experience. Nevertheless, the basic techniques are also available for many other languages, including English. In particular, we face two potential challenges: word form variance and compound words. German is a morphological complex language having a high variance in word forms expressed by many cases and inflections (cf. [34]). Looking at nouns, for example the word “Buch” (book) transforms to “Bücher” in the plural form or to “des Buches” for the genitive case. Regarding compound words, in German these are single words created by concatenating several words to a new word, e.g., “Fach|gruppe” (professional group).

In order to address these two challenges, two techniques from the natural language processing (NLP) area have been proven beneficial: stemming and word decomposition [8]. Stemming refers to the reduction of derived word forms to a common stem, e.g., “Grupp” for “Gruppe” and “Gruppen”. In the implementation of our approach, we use the stemming functionality of the Apache Lucene project^4^. For the decomposition of compound words, we use a language independent, lexicon-based approach developed by Abels and Hahn [1]. It generates possible splittings of words and checks whether the generated parts are covered in a lexicon. In our approach, we use JWordSplitter, an open-source implementation of this approach with an integrated German lexicon^5^.

The actual matching consists of two steps. First, we conduct a simple string match, and second, we decompose the business objects into their smallest semantic components and compare these with one another. The comparison of decomposed word parts is done by comparing the word stems. In this way, we are able to relate words such as “Fachgruppe” (professional group) and “Skillgruppen” (skill groups). The result of the described steps is an automatically provided list of potential activity-to-event-class relations on type level ($AE''$). An example of how our technique applies to the example of Fig. 1 is depicted in Fig. 4. The linguistic connections bridging the activity names with the descriptions and then connecting business objects with events are put in evidence by connecting dashed lines. The analyzed terms are highlighted. From the figure, it can be seen that the description of “Incident logging” mentions the need to: “assign the affected person” and person is the object of the event “Person added”. The same holds for the object “CI” in “Initial diagnosis” and “CI selected”, and for “KB” in “Incident closure” and “KB update performed”.

### Selection of the correct mapping

The integrated approach aims at combining different approaches for the matching of events and activities on type level. Therefore, we concurrently generate two sets of potential activity-to-event-class relations, namely $AE'$ and $AE''$. The generation of two sets originates from the insight that different approaches for the type-level matching vary in terms of coverage with respect to a final mapping. That is, for some approaches the set of potential activity-to-event-class relations may not include all relations required for the final mapping. Looking at the previously introduced type-level matching approaches that are based on Declare rules, it can be seen that these are designed to always include the complete final relations of activities and event classes in their potential activity-to-event-class relation [3]. This is due the fact that the approach based on behavior starts from all possible relations and prunes these relations by eliminating impossible combinations. If the assumptions made by this approach are fulfilled, the correct relation is always included in the set of potential relations. For the label analysis approach, this cannot be taken for granted. The label analysis approach starts with an empty set and adds those relations that can be found over the matching of extracted business objects. It may happen that not all relations of event classes and activities can be found.

As depicted in Fig. 3, both potential activity-to-event-class relations serve as an input for the selection of the correct mapping. The previous section introduced the approach for the automatic matching of event labels and activities. While it is rather obvious that the label analysis may lead to multiple mappings for one event class, we first discuss why there are often multiple solutions to the defined constraint satisfaction problem that is built based on Declare rules. From this background, we introduce means to guide the user through the set of potential mappings returned by the CSP solver and integrate the results from the label analysis into this approach.

**Fig. 5:**
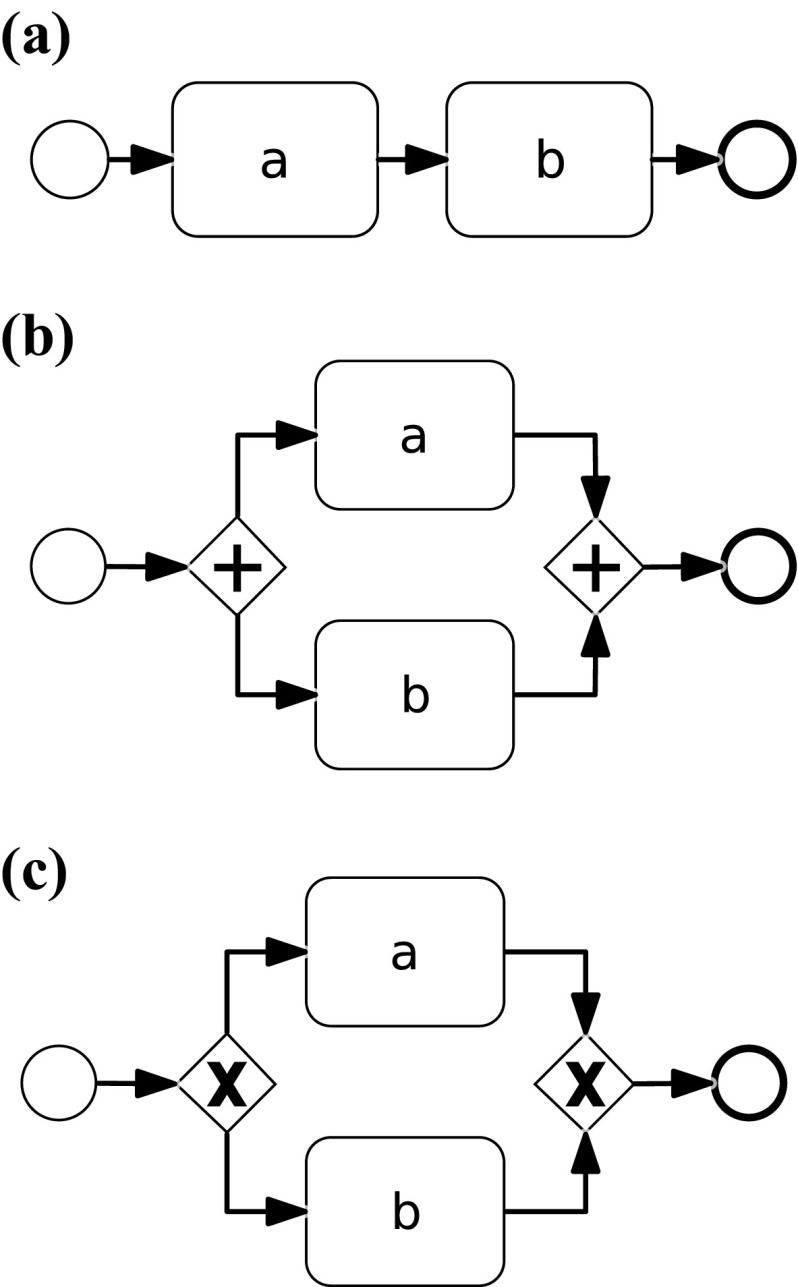
Process model fragments leading to multiple solutions of the Declare-based matching. **a** Sequence, **b** concurrency, **c** choice

Consider the trace $$t_1 = [k, l, m, n]$$ and the simple sequence of activities *a* and *b* shown in Fig. 5a, assuming that the log is not noisy. When matching $$t_1$$ and the sequence model, the corresponding CSP returns three solutions. In all three solutions, *k* is matched to *a* and *n* is matched to *b*. For *l* and *m*, it cannot be said whether they belong to *a* or *b* without further knowledge. It may be that both belong to *a*, or both belong to *b*, or *l* belongs to *a* and *m* belongs to *b*. The only mapping that can be excluded is that *l* belongs to *b* and *m* belongs to *a* at the same time. This is because from the event log consisting of only $$t_1$$, the following rules are discovered among the others: (i) $$ Init ( k )$$; (ii) $$ End ( n )$$; (iii) $$ ChainSuccession ( l , m )$$. The following rules are among the ones inferred from the model: (i) $$ Init ( a )$$; (ii) $$ End ( b )$$; (iii) $$ ChainSuccession ( a , b )$$. Due to constraints 5 and 6, an acceptable solution is such that $$Map\left( k \right) = a $$ and $$Map\left( n \right) = b $$. Because the mapping is 1:N for activities and events, it links one activity to one or more events, but not the other way around. Therefore, the acceptability of such solutions exclude that $$ a $$ and $$ b $$ are mapped to any other event. Events $$ l $$ and $$ m $$ *cannot* be mapped to $$ b $$ and $$ a $$, respectively. Indeed, we have that $$ ChainSuccession ( l , m )$$ holds true in the event log. If $$Map\left( l \right) = b $$ and $$Map\left( m \right) = a $$, then the premise of formula 9 is verified. However, $$ a $$ and $$ b $$ are not interleaving, because $$ ChainSuccession ( a , b )$$ holds true. On the contrary, $$ ChainSuccession ( b , a )$$ does not. Therefore, the consequent of formula 9 evaluates to false, whereas its antecedent is true. In turn, this means that constraint 9 (and, a fortiori, constraint 1) is violated; hence, no solution can map $$ l $$ to $$ b $$, respectively. By the same line of reasoning, $$ m $$ cannot be mapped to $$ b $$.

For Fig. 5b, we consider a log consisting of $$t_1 = [ k, l, m, n ]$$ and $$t_2 = [ n, m, l, k ]$$. If we want to match that log to the model shown in Fig. 5b, actually every combination of mappings is possible, except those where all events are mapped to only one of the activities. Indeed, the only rules from the list of Table 3 that hold true in the log are $$ Participation $$ for every event (e.g., $$ Participation ( k )$$), and $$ CoExistence $$ between each event and any other (e.g., $$ CoExistence ( k , l )$$, $$ CoExistence ( k , m )$$, $$ CoExistence ( k , n )$$, ...). The same holds for activities $$ a $$ and $$ b $$. As a consequence, only constraints like 7 and 4 are in the resulting CSP.

For the matching with the process model depicted in Fig. 5c, we add a trace to the aforementioned example log, henceforth consisting of $$t_1 = [ k, l, m, n ]$$, $$t_2 = [ n, m, l, k ]$$ and $$t_3 = [p, q, r, s]$$ . In this case, the CSP returns two solutions: Either every event in the set $$\{k, l, m, n\}$$ belongs to activity *a* and every event in the set $$\{p, q, r, s\}$$ to *b*, or the other way around. This is due to the fact that the $$ NotCoExistence $$ rules between every element of the first set and any element of the second one hold true in the log (e.g., $$ NotCoExistence ( k , p )$$, $$ NotCoExistence ( k , r )$$, $$ NotCoExistence ( q , p )$$, ...). The rule $$ NotCoExistence ( a , b )$$ is inferred from the model. Therefore if, e.g., $$ k $$ and $$ l $$ were mapped to $$ a $$ and $$ b $$, respectively, this would contradict constraint 8 (and, a fortiori, 4).

Such ambiguous mappings, i.e., cases in which the CSP has multiple solutions, cannot be automatically resolved and require a domain expert to elect the mapping for the concerned events and activities. Nonetheless, this decision can be supported by the mapping approach. To aid the analyst with the disambiguation of multiple potential mappings, we introduce a questioning approach, which is inspired by the work of La Rosa et al. [50]: The user is guided through the configuration of a process model using a questionnaire procedure. The analyst is presented one event label at a time along with the possible activities to which this event label can be mapped. Once the analyst decides which of the candidate activities belongs to the event label, this mapping is converted into a new constraint that is added to the CSP. Consecutively, the CSP is solved again. In case there are still multiple solutions, the analyst is asked to make another decision for a different event label. This procedure is repeated until the CSP yields a single solution. The goal is to pose as few questions to the analyst as possible. To achieve this goal, we look into all solutions and choose the event label that is assigned to the highest number of different activities. Notice that the order of the selection influences the efficiency of deriving the single solution. By selecting the event that is related to the highest number of activities over all solutions, we aim at striking out the highest number of wrong mappings in each iteration. Thereby, the efficiency is improved. Effectiveness is instead not influenced by the order of selection.

We use the relation $AE''$ as a filter when presenting the activities between which the analyst has to choose. In case an event class $e$ is mapped to multiple activities over all relations contained in the base relation $AE'$, the analyst has to inspect which of these multiple activities are correct mappings. Having both relation $AE'$ and $AE''$, the analyst will only be presented the activities that have a mapping to event class $e$ in both relations. We denote the set of activities potentially mapped to event class $e$ in the base relation as ${ A}_{e}' = \left\{ a_{e}' \, | \, \exists (a_{e}', e) \in AE' \right\}$. Similarly, the derived activities for $e$ contained in the filter relation are denoted as ${ A}_{e}'' = \left\{ a_{e}'' \, | \, \exists (a_{e}'', e) \in AE'' \right\}$. The set of presented activities for event class $e$ is defined as ${ A}_{e}^* = { A}_{e}' \cap { A}_{e}''$.

Due to the fact that the relation $$AE''$$ may not contain the correct mapping, it can happen that also $${ A}_{e}^*$$ does not contain the correct matching activities for event class $$e$$. Therefore, the analyst can indicate that there are missing matches. Consequently, a new set of activities is presented from which set the analyst can complete their choice. This second set of activities is defined as $${ A}_{e}^{**} = { A}_{e}' \setminus { A}_{e}''$$ and contains only those activities found in a relation to event class $$e$$ in $${ A}_{e}'$$. As it holds that $${ A}_{e}' = { A}_{e}^* \cup { A}_{e}^{**}$$, the correct activities have to be contained in the two presented sets. By splitting the set of activities that an analyst has to inspect, the selection step is made easier as less information has to be processed at the same time.

Once a decision on the final mapping is made, the user can annotate the relations between event classes and activities with a transition life cycle, namely the phase that the occurrence of the event characterizes within the enactment of the activity. The starting and ending transitions are not required to be specified, because they will be automatically detected in the subsequent phase.

### Transformation and activity instance clustering

Having defined the procedure to build a CSP and iteratively resolved any ambiguities, the next step is to use the selected solution of the CSP as mapping Map to transform the event log. Mapping Map is used to iterate over all traces in the event log and replace each event ${e_i}$ with the activity returned by $Map({e_i})$.

Having mapped all event instances to the life-cycle transitions of their corresponding activity type, the subsequent step is to define how to assign events belonging to the same activity to different activity instances. As there might be multiple activity instances for one activity in a process instance, i.e., in a loop, criteria to map an event to an activity instance are required. To this extent, we adopt the technique detailed in [5]. The user specifies the so-called instance border conditions, discriminating between events belonging to two or more instances of the same activity. Instance borders can be also defined over any attributes attached to an event. Having this information, the traces of the preprocessed event log where all event instances are mapped to their corresponding activity are iterated through. A tree-based incremental clustering algorithm known from classical data mining is used [59] to assign events to different activity instances. The first and the last events of a cluster are assigned the “Start” and “Complete” transition, respectively. The events in between are assigned the life-cycle transitions indicated by the user in the previous step. Further details on the adopted technique and on the instance clustering algorithm are provided in [4].

The transformed event log can then be used as an input for any process mining technique.

## Validation and evaluation

In this section, we will present the results from our validation and evaluation. Section 4.1 provides the details of the validation and evaluation setup that we have chosen. In Sect. 4.2, we validate the introduced Declare approach with synthetic event logs derived from a real-life industry process model collection. We inspect and outline the different influences of certain constraints that have been introduced for special cases. We report on an industry case study for the integrated approach in Sect. 4.3 and finally, discuss shortcomings and future work in Sect. 4.4.

### Validation and evaluation setup

**Fig. 6:**
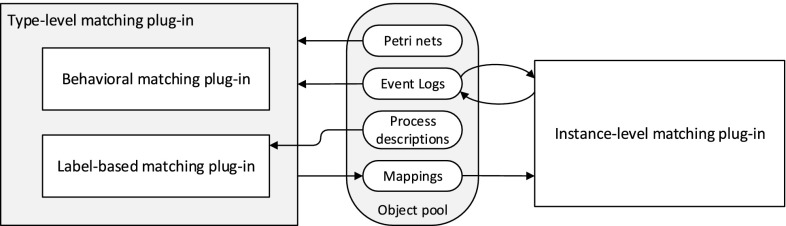
FMC Block diagram of the implemented ProM plug-ins with inputs and outputs

**Fig. 7:**
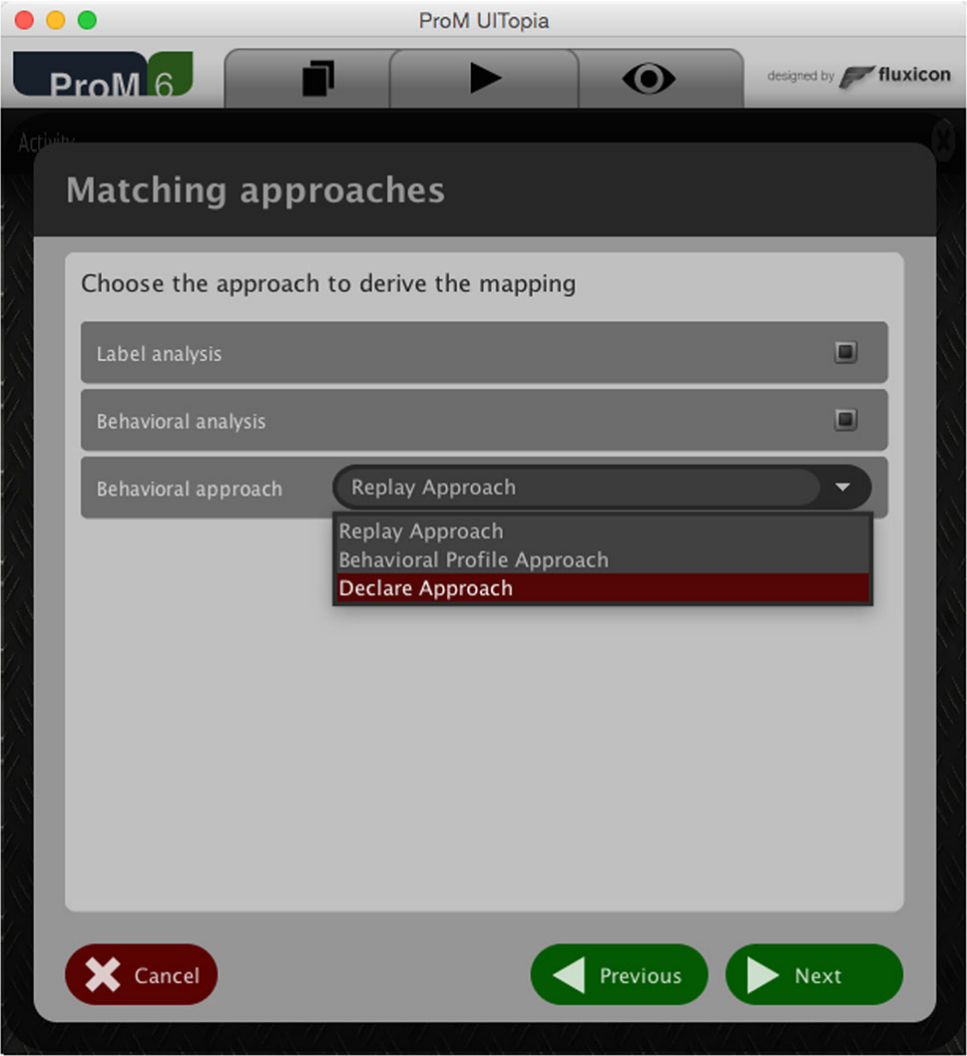
Configuration screen for the type-level mapping plug-in

For the purpose of evaluation, we implemented the introduced approach for the matching of events and activities in the ProM framework^6^. All plug-ins that have been developed for the evaluation of the concepts introduced in this paper can be found in the publicly available ProM package “Event2ActivityMatcher”^7^. Figure 6 depicts a FMC Block diagram^8^ that gives an overview of the implemented ProM plug-ins. The mandatory inputs for the type-level mapping plug-in are an event log and a Petri net. Optionally, a process description, which can be used by the label analysis approach, may be provided. Both the label analysis and the Declare approach are implemented as separate plug-ins to make them independently usable. The type-level plug-in provides a configuration screen to choose between the different mapping approaches and provides the capabilities for their integration. The first configuration screen of the type-level plug-in is shown in Fig. 7. Note that this plug-in also supports the use of the replay approach and the behavioral profile approach introduced in [6] and [7]. Yet, both approaches only support one-to–one mappings.

In order to evaluate the introduced concepts, we have conducted both a validation of the Declare approach using synthetic data, and a case study with real-life data from a large German outsourcing company. The label analysis approach had previously been evaluated using two case studies that highlighted its effectiveness [5].

The goal of the validation is to assess (1) the *effectiveness* and (2) the *efficiency* of the Declare approach. By effectiveness, we mean the ability to derive the correct mapping. With efficiency, we refer to the necessary effort in terms of manual work. Furthermore, (3) the *robustness toward noise* and (4) the *performance* of the approaches shall be evaluated.

In order to measure (1) the effectiveness of the approaches, we evaluate whether the correct mapping can be retrieved within a reasonable time frame. Looking at (2), the efficiency, we quantify the manual work by counting the questions an analyst has to answer in order to arrive at the final mapping. The underlying idea is that users are most likely going to perceive the burden of the time spent when they are actively involved and requested to answer questions. Owing to this, we assume as a basic metric the number of asked questions. We acknowledge that this estimation disregards how difficult it is for users to reply to such questions in terms of mental effort. This limitation is due to practical reasons: The effort would indeed vary from case to case and depend on the experience of the analyst with the data at hand. The robustness toward noise (3) is evaluated by generating five different event logs for each process model with increasing levels of noise. For each process model, one event log with 1000 traces is simulated using the simulation technique provided by [49]. These noise-free event logs serve as a base to generate noisy event logs by randomly applying different noise patterns to a fraction of the traces. The noise patterns refer to the shuffling, duplication and removal of events. In this way, we produce five event logs for each process, each having different amounts of traces affected by noise, namely: (1) 0% (no noise), (2) 25%, (3) 50%, (4) 75%, and (5) 100%.

**Fig. 8:**
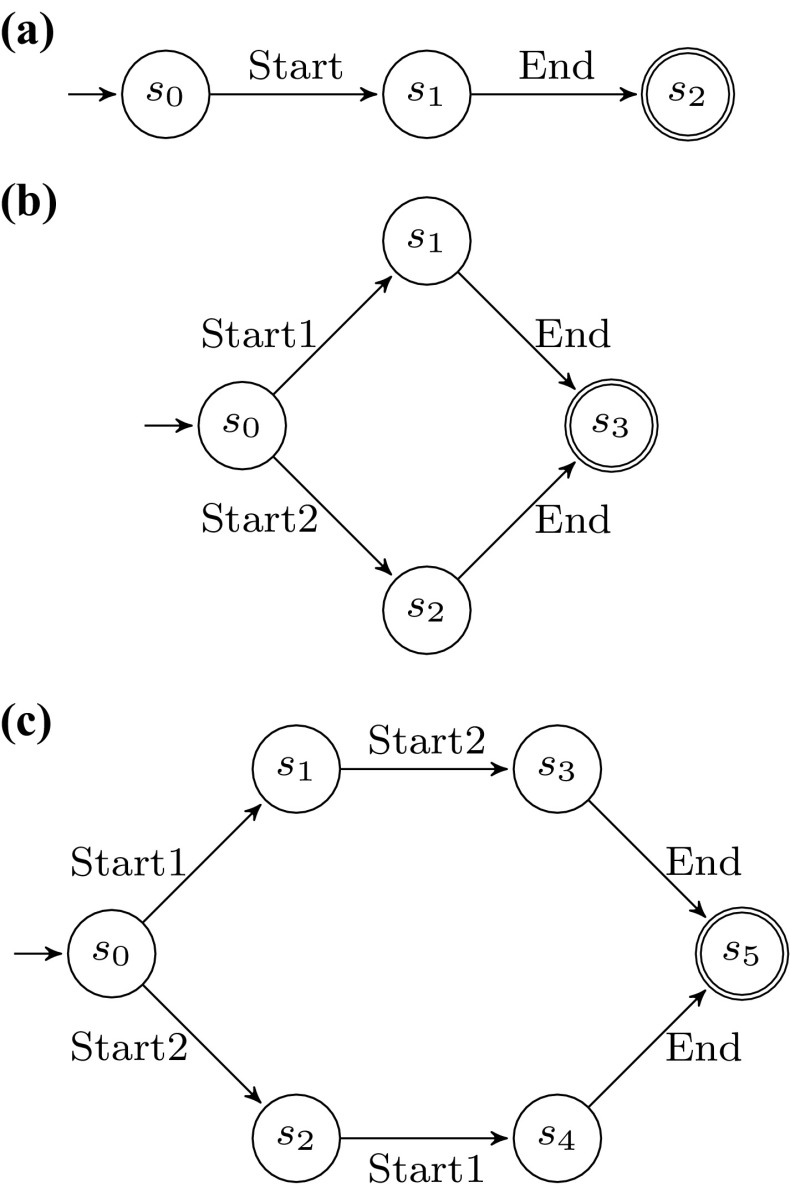
Different event models used to generate events. **a** Sequence of start and end events. **b** Two alternative start events, one end event. **c** Two concurrent start events, one end event

In order to evaluate the handling of different abstraction levels, event logs were generated by simulating the enactment of process activities through event generators. Such event generators simulate a simple activity life-cycle model containing a start and a complete life-cycle transition. We chose three different event patterns that can be mapped to such a life-cycle model based on the process instantiation patterns introduced by Decker and Mendling in [12]. Figure 8 depicts the different chosen patterns. Figure 8a shows a simple model with one start and one end transition (“Start” and “End” events), demonstrating a typical pattern found in many systems. For each activity assigned to this event model, a start and an end transitions are generated for each execution of that activity. The second event model, depicted in Fig. 8b, generates for each execution either an event “Start1” or an event “Start2” and always an “End” event. Thus, there are two alternative starts for such an activity, e.g., it could be started by an incoming mail or by a telephone call. The event model presented in Fig. 8c also has two different start transitions, but in contrast to the model in Fig. 8b, both start events always occur with no restriction on their order. For the simulation of the process models, each activity is randomly assigned to one of these three event models, or it is left as is, generating only a single event. Again, all generated event logs contain 1,000 traces and are limited to 1,000 events per trace as a stop condition for process models containing loops.

All experiments were conducted in a cluster environment where each matching experiment was assigned 6 Gigabytes of main memory and 4 CPU cores running at 2.93 GHz. This reflects the processing power of a typical desktop machine these days. For each experiment, a timeout of 10 min had been set, after which the experiment was terminated if the constraint satisfaction problem was not yet solved. Basing upon the experimental results of [18, 22], we have set the default threshold for the minimum support of discovered Declare rules to $90\%$.

The set of business processes used for the validation of our work on matching approaches using Declare rules stems from the *BIT process library, Release 2009*, which has been analyzed by Fahland et al. [27] and is openly available to academic research. The process model collection contains models of financial services, telecommunications, and other domains. The models are real-life process models that have been anonymized to make them available for research.

The BIT process library is separated into five groups of process models: A, B1, B2, B3, and C. Of these groups, B1, B2, and B3 contain different versions of the same models created at different points in time, with B3 incorporating the latest versions [27]. Therefore, we only use the process models from groups A, B3, and C. In the further process of our evaluation, we will not distinguish between these three groups.

Finally, we also removed all process models that only contain a single activity, because matching is trivial in such a case. After applying all of the described filtering steps, 442 models remain and are used for the evaluation of our behavioral approaches. From these models, two sets of event logs were generated. One set reflects the one-to-one setting for which simple simulation has been used. The other set contains event logs on a lower abstraction level, created by using the aforementioned event generation patterns. Both sets contain 2,210 event logs each (442 models times 5 noise levels).

For the evaluation of the integrated approach on real-world data, we conducted a case study with a large German IT outsourcing provider and analyzed the process of managing standard changes, which is part of the change management process defined by the IT Infrastructure Library (ITIL). The process is supported by an IBM Tivoli Change and Configuration Management Database^9^ from which we extracted a log containing 364 traces with 5194 event instances of 14 different event classes. The corresponding process model contains seven activities that are further detailed with activity descriptions from a work instruction document.

### Validation of the Declare-based type-level matching

For the validation of the Declare-based type-level matching, we inspect different configurations for the approach in order to assess the influence of different constraints. We define a *basic* configuration, which does not include constraints from interleaving relations and does not use the relaxed mapping for ordering and co-occurrence relations. Next, we define five different configurations that are all based on the basic setting. Constraints stemming from interleaving relations are added in the *interleaving* configuration. We exclude participation constraints in the *no participation* configuration. The *relaxed co-occurrence* and *relaxed ordering* configurations use the relaxed definition of the respective constraints. The last configuration is a combination of the already defined configurations but leaves out the interleaving constraints. Hence, it is called *all but interleaving*.

#### Effectiveness: one-to-one setting

**Fig. 9:**
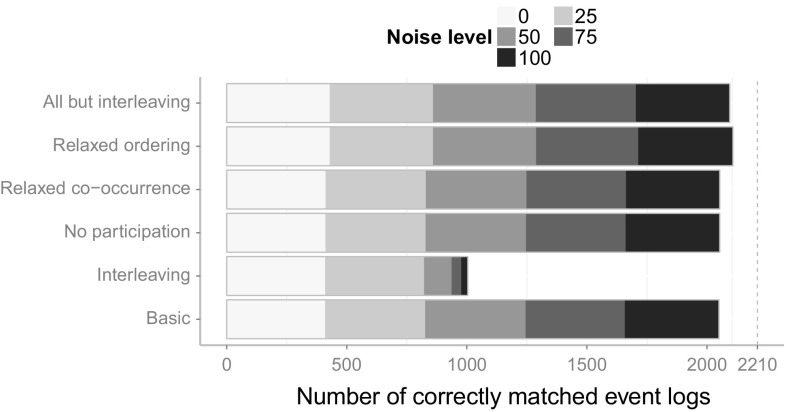
Declare approach: number of correctly solved matchings in a one-to-one setting for different noise levels

Starting with the *effectiveness*, Fig. 9 shows for each configuration how many event logs could be successfully matched to their corresponding process models on type level. The figure separates the event logs by their noise level. It can be seen there are only minor differences between most of the configurations. The majority of configurations is able to correctly solve 93–95% of all matchings. Only the *interleaving* configuration scores low, with 45% correct matchings. Figure 9 reveals the main problem of the configuration with constraints stemming from interleaving relations: It cannot deal with noise levels above 25%. The reason for this is that with increasing noise, less order relations reach the minimum support and therefore less order constraints are created. These relations are not seen as order relations anymore and are rather interpreted as interleaving relations, thus resulting in conflicting constraints.

Overall, the *relaxed ordering* and the *all but interleaving* configuration score highest with 95% correctly solved mappings.

**Fig. 10:**
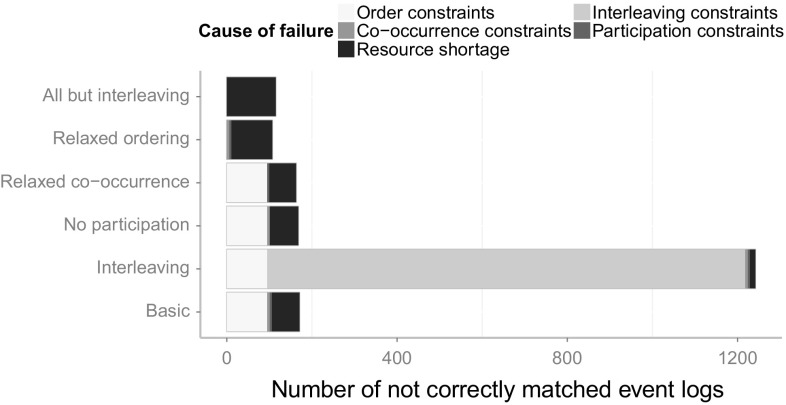
Declare approach: number of not correctly matched event logs in the one-to-one setting (all noise levels)

Figure 10 sheds light on the reasons for incorrect mappings. We drill down to the specific types of constraints that were pushed into the constraint satisfaction problem. Besides the conflicting interleaving constraints in noisy event logs, also constraints stemming from order relations lead to problems in the matching. For all but the top two configurations, around 5% of the matchings cannot be solved correctly due to wrong constraints stemming from order relations. The conflicts stem from interleaving activities for which their corresponding events show a dominant ordering. That means that two events that could potentially occur in any order are seen almost always in the same order. These wrong constraints can be resolved by employing the relaxed mapping for ordering relations. Hence, the *relaxed ordering* and *all but interleaving* constraints do not contain any incorrect order constraints.

In a similar way as the ordering constraints, also the co-occurrence constraints suffer from dominant behavior in the event log. Here, the root cause lies in optional activities that are executed in a dominant fashion, i.e., they are present in almost all cases. In the simulated data, this happened only for the event logs of one process and is resolved by using relaxed co-occurrence constraints.

Figure 10 reveals that there are cases that cannot be solved due to computational resources shortage. It can be seen that the number of cases with computational resource shortage decreases when additional constraints from interleaving relations come into play. On the contrary, the number of cases with computation issues increases when constraints are relaxed. While not using or relaxing certain constraints removes conflicts that prevent the correct mapping, it comes at the price of higher computational effort as the search space grows. If a process can be solved or not with a certain configuration, heavily depends on the structure of the process and on the characteristics of the event log. A deeper analysis revealed that processes with a high degree of concurrency often lead to computational resources shortage.

#### Efficiency: one-to-one setting

**Fig. 11:**
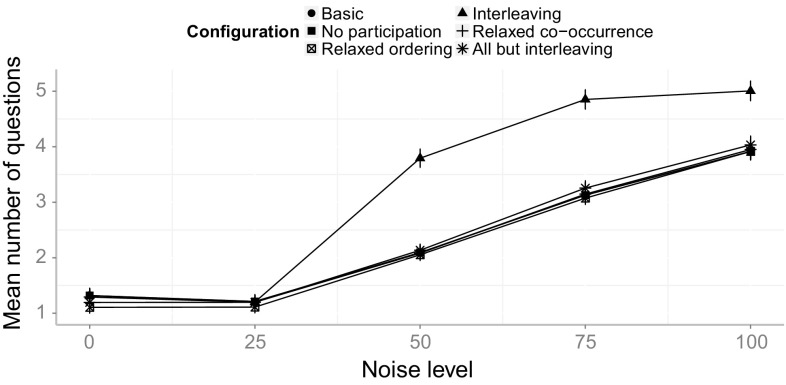
Declare approach: mean number of questions for each configuration

In order to assess the *efficiency*, we measured the mean number of questions that had to be asked for each configuration and each noise level, as depicted in Fig. 11. In those cases in which noise was not injected or involved 25% of traces, the data show that all configurations result in a similar mean number of questions ranging from 1.1 to 1.32 with *relaxed ordering* scoring best. For all noise levels above 25%, the number of questions increases for all configurations. Still, almost all configurations behave very similarly with a steady increase in one question on average. Only the *interleaving* configuration requires significantly more questions. From the results gathered for the effectiveness (Sect. 4.2.1), it is known that the *interleaving* configuration is not able to solve many mappings for event logs with noise levels above 25%. For those cases the maximum number of questions, i.e., one question for each event class, has to be asked.

**Fig. 12:**
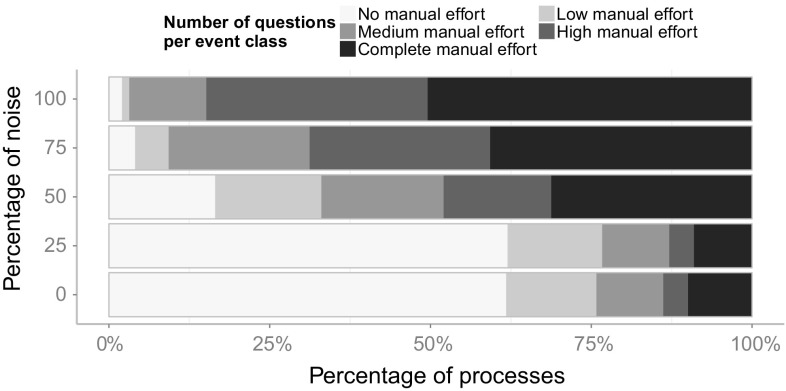
Declare approach—basic configuration: number of questions per event class for each noise level in one-to-one setting

As none of the configurations shows a statistically significant advantage over all the others, we will use the *basic* configuration for our further analysis. Figure 12 depicts the number of required questions relative to the number of event classes for the *basic* configuration. The share of mappings that could be performed completely automatically is 62% of all cases with a noise level below 50%. For 14–15% of all cases with less than 50% noisy traces one question for at most every fourth event class (low manual effort) is required. Another 10% of the cases with a noise level below 50% could be matched with medium manual effort, i.e., with at most one question for every second event class. Summing this up, 86–87% of all cases with a noise level below 50% could be solved with at most medium effort with the *basic* configuration. Looking at the share of event logs for which a complete manual mapping is required, only 9–10% of all event logs with less than 50% noisy traces are left completely to the analyst.

**Fig. 13:**
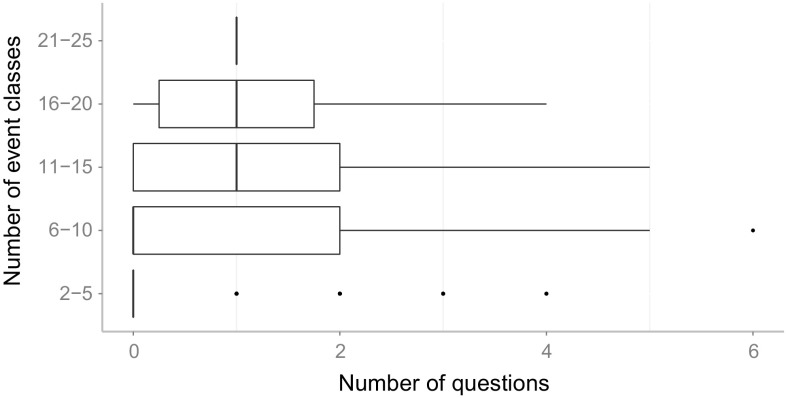
Declare approach—basic configuration: number of questions per event class for correctly matched event logs without noise in one-to-one setting

Changing the perspective of the analysis to the number of event classes contained in an event log, Fig. 13 inspects all event logs without noise and divides the event logs by their number of event classes into five categories. For each category, a box plot for the required number of questions is shown. For event logs with up to five event classes, the Declare approach runs fully automatically for almost all cases. For the category of six to ten event classes, half of the cases are handled without a question. For all other categories, 50% are matched automatically. Notably, there is no linear increase in the number of questions with growing numbers of event classes. This shows that especially larger event logs profit from the introduced reduction technique.

#### Robustness: one-to-one setting

With respect to the *robustness toward noise*, Figs. 9, 10, 11 and 12 already provide insights on how the effectiveness and efficiency of the Declare approach change with increasing noise in the event logs. Regarding the effectiveness, Fig. 9 reveals that all configurations except the *interleaving* one are very robust toward noise.

Concerning the efficiency, the Declare approach proves to be stable only until a noise level of 25%. Beyond this level of noise, the efficiency drops down. The approach is still able to handle in a completely automated way 17% of the event logs in which every second trace contains noise. Overall, the Declare approach is still helpful for 70% of the event logs with 50% of noise and out of these it handles 76% with at most medium effort. With three quarters of the event logs containing noise, efficiency drops again. Nevertheless, even with all traces containing noise, the Declare approach is still helpful for 50% of the event logs.

#### Performance: one-to-one setting

**Fig. 14:**
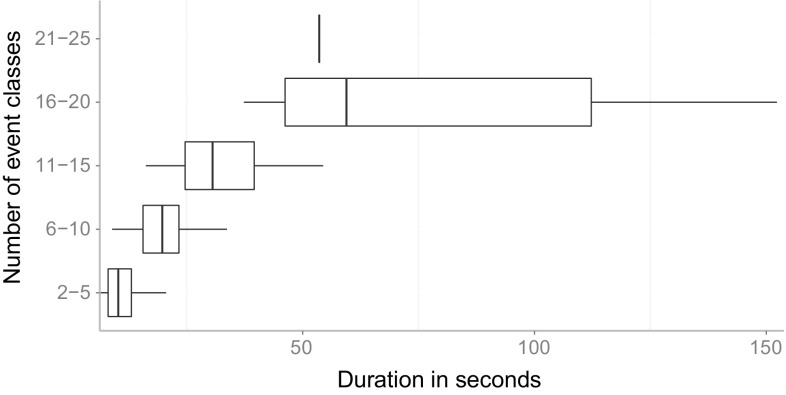
Declare approach—basic configuration: duration of the matching depending on the number of event classes in one-to-one setting without noise (without outliers)

Turning to the *performance* of the Declare approach, Fig. 14 depicts how long the matching takes depending on the number of event classes in the event log. For the group with the fewest event classes, the basic setting requires less than about 10 s for half of the matchings. The 0.75 quantile lies at 13 s. With increasing number of event classes, the duration of the matching increases almost linearly. For the event logs with up to 20 event classes, the median lies around 1 min, but cases my take up to more than 2 min. Still, we believe that this is fast enough for the one time undertaking of the type-level matching.

#### Effectiveness: one-to-many setting

**Fig. 15:**
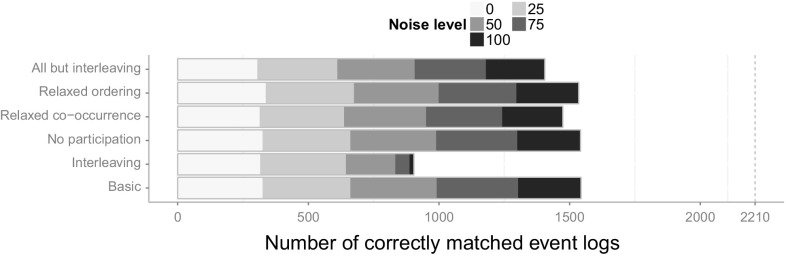
Declare approach: number of correctly solved matchings in one-to-many setting for different noise levels

Turning to the one-to-many setting, Fig. 15 provides the results for the measurement of the *effectiveness*. While the overall pattern looks very similar to the one seen for the one-to-one setting (Sect. 4.2.1), there is a visible difference in the overall number of event logs that can be matched correctly. For the one-to-many setting, the maximum number of correctly matched event logs over all noise levels is 1544, which is 70% of all 2210 event logs. This is 25% points less than what could be handled in the one-to-one setting. It can be seen that effectiveness slightly decreases for all configurations with increasing noise. Without noise, the most effective configuration is the relaxed ordering configuration, which correctly maps 76% of all noise-free event logs.

**Fig. 16:**
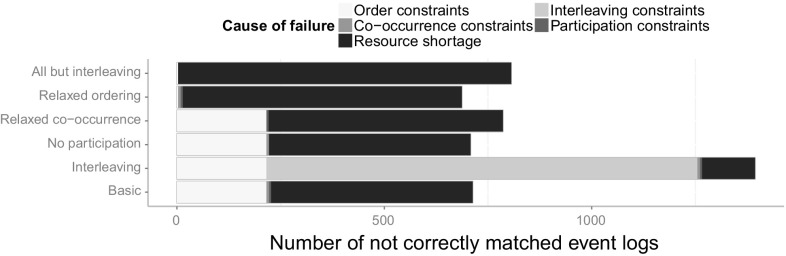
Declare approach: number of not correctly matched event logs in the one-to-many setting (all noise levels)

Analyzing the root causes for the decrease in effectiveness, Fig. 16 reveals that most of matchings cannot be solved due to computational resources shortage. Besides that, the same root causes that were discovered in the one-to-one setting also apply in the one-to-many setting. While the impact for almost all constraint types stays the same, the number of cases that cannot be matched due to wrong order constraints doubles in the one-to-many setting.

#### Efficiency: one-to-many setting

**Fig. 17:**
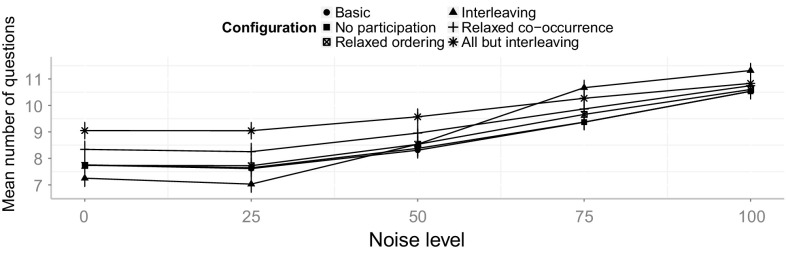
Declare approach: mean number of questions for each configuration in the one-to-many setting

Bringing the focus to the *efficiency*, Fig. 17 depicts the mean number of questions for each noise level and every selected configuration. In contrast to the one-to-one setting, one can observe more distinct differences between the configurations in the one-to-many setting. Again, the interleaving configuration performs worst for high noise levels. Yet, it outperforms all other configurations for lower noise levels. Looking at the ranges in which the average number of questions lies, it can be observed that these are higher than those for the one-to-one setting, which one would expect. Overall, the mean numbers of questions range between seven and nine for lower noise levels and go up to eleven questions on average for the highest noise level.

**Fig. 18:**
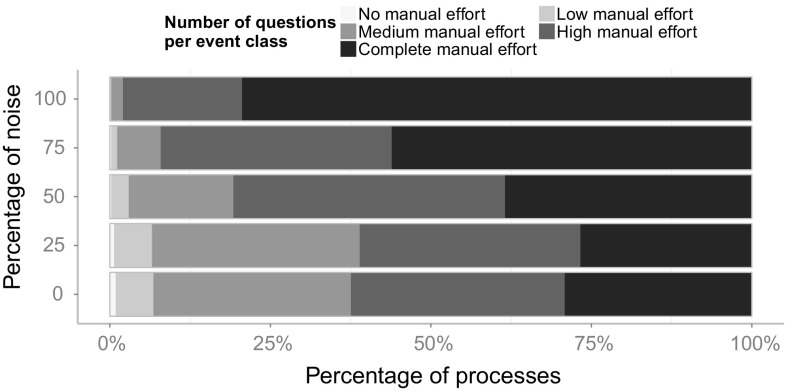
Declare approach—basic configuration: number of questions per event class for each noise level in one-to-many setting

The relative view of number of questions per event class is given in Fig. 18. Only very few event logs can be processed completely automatically (1% of the event logs with low noise levels). However, with at most medium manual effort 38–39% of the event logs with noise level zero and 25 can be matched. Again, the small noise level helps in getting rid of incorrect ordering relations and therefore the approach performs better for these event logs than for logs that are noise-free. Overall, it can be observed that the approach is helpful for 70–72% of the event logs with no or few noise insertions, which again is still the majority of those event logs. Yet, with increasing noise the efficiency shrinks.

**Fig. 19:**
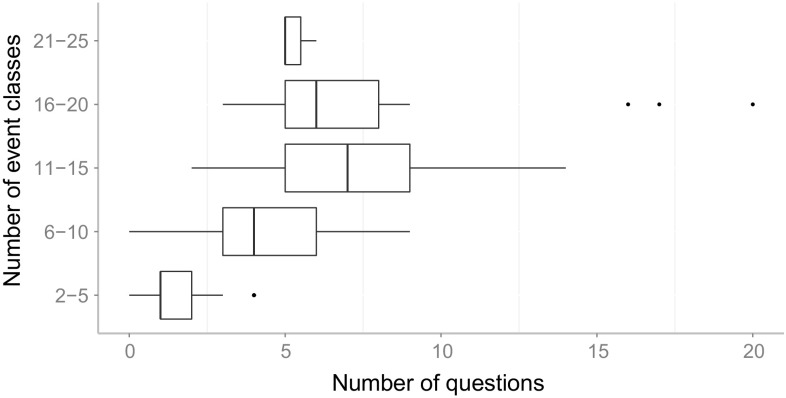
Declare approach—basic configuration: number of questions per event class for correctly matched event logs without noise in one-to-many setting

The influence of the number of event classes in an event log is studied in Fig. 19. In the one-to-many setting, the differences between the five categories become more distinct. First, one can observe an increase in the number of questions with a growing number of event classes. Yet, this development is turned around for logs with more than 16 event classes. Here, a slight decrease can be seen. Looking at event logs with 16 to 20 event classes, only at most six questions are required for half of the event logs, whereas at most seven questions are required for half of the event logs with 11 to 15 event classes.

#### Robustness: one-to-many setting

Coming to *the robustness toward noise*, we can again use the insights already provided during the analysis of effectiveness and efficiency. From Fig. 18, it can be seen that there is a slight increase in the number of matchings for which the approach is useful when there is a small amount of noise in the event logs compared to when no noise is present. From the noise level of 50% upwards, this number constantly decreases until there is only a share of 21% for which the behavioral profile approach helps the analyst. Overall, this development is very similar to that observed in the one-to-one setting, yet, on a much lower order of magnitude and with steeper decrease in effectiveness and efficiency with higher noise levels.

#### Performance: one-to-many setting

**Fig. 20:**
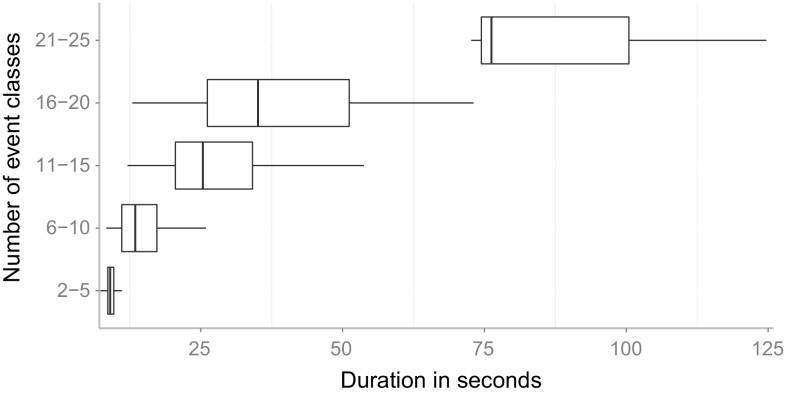
Declare approach—basic configuration: duration of the matching depending on the number of event classes in one-to-many setting without noise (without outliers)

Looking at the performance in the one-to-many setting, Fig. 20 shows the box plots for the matching durations for the introduced categories of event classes. Again, the duration increases with a growing number of event classes. Yet, this time the growth rate is worse than linear. Nonetheless, with less than 10 s for the vast majority of the smallest category and less than 2 min for majority of the largest event logs, the performance seems to be reasonably good.

### Evaluation of the integrated approach

The first steps in the integrated approach are formed by the concurrent creation of the two potential activity event class relations. From these two relations, the one created by the Declare-based approach is used as base relation, while the other one produced by the label analysis approach is used as a filter relation.

Starting with the results for *(1) the effectiveness* of the integrated approach, we concentrate on the creation of the base relation, because this relation is the critical element, that is, if and only if such relation contains the correct mapping, the approach can effectively solve the matching. We therefore assess which configuration of the behavioral approaches is able to solve the matching correctly. In order to do this, the process manager provided a manual mapping that serves as gold standard, against which we check whether the derived type-level mapping is correct or not. If it is not correct, we determine the constraints that are conflicting with the gold standard mapping.

Two configurations of the Declare-based approach do not contain any wrong constraints and are therefore able to solve the CSP. All other configurations fail due to twelve incorrect co-occurrence constraints. The reason for this is that there is one optional activity in the standard change process model for which event instances of one of the corresponding event classes are almost always present. This event class represents the final measuring of time taken and belongs to an optional quality assurance activity. As this time measurement is performed in almost every case, co-occurrence relations are derived with all event classes for which we also almost always see their event instances. That is, event instances belonging to mandatory activities. Nonetheless, these co-occurrence relations do not exist in the process model since the quality assurance activity is optional and therefore is not part of any co-occurrence relation.

With the two configurations *relaxed co-occurrence* and *all but interleaving*, the integrated approach can be successfully applied. We will therefore proceed with these two configurations and turn to the analysis of *(2) the efficiency*. Both configurations lead to the same single question. The one event class for which the analyst needs to decide the mapping activity can potentially belong to every activity. That is, the user is presented all seven activities of the process model to choose from.

Yet, in the integrated approach, these seven activities are filtered using the potential activity event class relations derived by the label analysis approach. An extensive evaluation of the label analysis approach alone has been provided in [5], where the change management process is also analyzed. We refer the reader to [5] for a detailed assessment. The relation derived from the label analysis contains only two matching activities for the event class in question. Due to the very good recall of the label analysis approach, which is 86% for the standard change process, the correct activity is contained. Therefore, only two activities need to be presented to the user, which is a substantial decrease from the seven activities that the independent Declare-based approach would have to present.

Finally, we turn to the inspection of *(3) performance* and *(4) robustness to noise*.

Regarding the performance of the integrated approach for type-level matching, we measured the required time until the first question is posed to the user. The Declare-based approach took around 40 s to solve the initial CSP. The label analysis approach took about 30 s to deliver the potential activity event relations, thus turning out to be a bit faster, but quite similar. As both approaches run in parallel, the time to wait until the first user interaction amounts to 40 s. As the type-level matching only needs to be conducted once, we believe this to be reasonably fast enough.

As we are looking at a real-life event log from an IT system where the designed process model is not enforced, it is very likely that the event log contains some behavior that is not specified by the process model. In order to inspect the amount of noise contained, we calculated the constraint-relative behavioral profile conformance metric introduced by Weidlich et al. [57]. For the preprocessed event log of the standard change process, an overall constraint-relative behavioral profile conformance of 91.87 is achieved. This proves that the filtered event log still contains noise, which is successfully handled by the integrated approach.

### Discussion and future remarks

In light of our experimental results, the Declare-based approach showed overall a good performance, especially with regard to resilience to noise. It requires in most of the cases only little manual intervention. Still, there are some processes that could not be handled, mainly due to massive parallelism and resulting memory shortage. Future work should investigate how these processes can be handled or, at least, automatically identified.

It is our plan to investigate how the approach can be extended to support N:M relations, namely cases in which a single event class can be related to multiple activities—e.g., events representing shared functionalities. In the N:M case, the already very large search space for the matching problem grows drastically and other techniques might be necessary to handle this.

Moreover, we plan to include further perspectives in the creation of a CSP—for example, by including roles from the organizational perspective. One could use the results of organizational mining or existing knowledge about the roles that are assigned to the users of an IT system to formulate further constraints. Such constraints could, for example, allow only mappings between events and activities that share the same executing role. When multiple IT systems are involved in the execution of a process, the knowledge about (i) which IT system supports which activity, and (ii) which event stems from which IT system could be used to generate further constraints. Also information on how control-flow routing is done could be integrated to retrieve further constraints. To this end, for example, decision tables could be leveraged to limit the number of activities to which an event class can potentially map.

To enhance the accuracy of the label-based matching, we will investigate how to exploit the inclusion in the analysis of the semantic relationships of used words, such as synonyms, hyponyms, and hypernyms. The idea is to find the connection of event and activity labels also when they do not share any stems, yet refer to concepts in the same field. For the automatic analysis, lexical databases such as WordNet [41] or BabelNet [42] are available. Previous studies exploiting the detection of semantic relationships between labels have been demonstrated successful among others in the fields of process models matching [37] and similarity measuring [25], as well as for the detection of lexical ambiguities within process models [46].

Experimental studies on the sensitivity of Declare rules to noise have demonstrated that the semantics of the rules have an impact on the decrease in their support in proportion to errors in the recorded traces [19, 22]. We will therefore analyze to what extent the support threshold can be adjusted depending on the singularly involved rules, in order to reduce the effect of misrecorded events on the outcome of the discovery phase.

Recent advances in the automated discovery of declarative processes have shown promising results when applied to branched Declare [14, 15]. Branched Declare allows the specification of rules that link the occurrence of an activity to the occurrence of multiple other ones. An example is ${{ Precedence }(\left\{ a , b \right\} , c )}$, stating that activity $c$ must be preceded by $a$ or $b$. Arguably, such an extension of standard Declare provides a richer expressive power, which can in turn help us improve the accuracy of the behavior-based mappings between activities and events. Therefore, we aim at integrating the existing branched Declare mining techniques with our approach in our future work.

Finally, we remark here that the process modeling notation here considered is BPMN. BPMN has a rich expressiveness also due to the availability of a plethora of advanced constructs, including intermediate events, exception flows, event-based exclusive choices, and more [24]. Such advanced elements could contribute to better identify discriminative patterns in event logs that we could exploit to our matching purposes. We will thus investigate how to extend our analysis taking into account more complex constructs of the BPMN specification.

## Related work

Related research can be subdivided into approaches working on event logs and approaches working on process models. Looking at approaches focusing on event logs, there are several ones aiming at the abstraction of events to activities. Günther et al. introduce in [29] an approach that clusters events to activities using a distance function based on time or sequence position. Due to performance issues with this approach, a new means of abstraction on the level of event classes is introduced by Günther et al. [31]. These event classes are clustered globally based on co-occurrence of related terms, yielding better performance but lower accuracy. A similar approach introducing semantic relatedness, N:M relations, and context dependence is defined by Li et al. [38]. Another approach that uses pattern recognition and machine learning techniques for abstraction is introduced by Cook et al. [11]. Together with the fuzzy miner, Günther and van der Aalst present an approach to abstract a mined process model by removing and clustering less frequent behavior [30]. While all these approaches aim at a mapping of events to activities, they are designed to automatically construct activities and not to match events to activities that have already been defined a priori. In [5] and [6], approaches that aim at the mapping of events to predefined activities are introduced. Nevertheless, the approach in [5] still required much manual work as the precision of matchings is not sufficiently high. In contrast, the approach presented in this paper requires only very little manual effort to match events to predefined activities. The approach presented in [6] only works with 1:1 relations between events and activities and requires preprocessing for 1:N relations. Furthermore, it is only able to capture behavior from traces that can be replayed on the model. This is resolved by the work of this paper.

Another branch of related approaches working on event logs are those dealing with event correlation to group events belonging to the same process instance, as e.g., the work by Perez et al. [44]. Yet, these approaches work on a more coarse-grained level as they focus on the relation to process instances rather than to activities. In fact, we assume that the correlation of events to process instances is either already given, or can be established by an approach like [44].

Our work is also related to automatic matching for process models. While matching has been partially addressed in various works on process similarity [23], there are only a few papers that cover this topic as their major focus. The work on the ICoP framework defines a generic approach for process model matching [55]. This framework is extended with semantic concepts and probabilistic optimization in [35, 37]. Further, general concepts from ontology matching are adopted in [26]. The implications of different abstraction levels for finding correspondences are covered in [54]. Recently, various approaches have been proposed and tested in the Process Model Matching Contests 2013 and 2015 [2, 10]. However, all these works focus on finding matches between two process models, not between events and activities.

## Conclusion

In this paper, we introduce a novel technique for the mapping of events to activities, which can be used as a preprocessing step to enable business process intelligence techniques (e.g., process mining). The approach uses Declare rules derived from existing business process models and from event logs generated by IT systems to establish a connection between conceptual process models and operational execution data. Event and activity labels as well as existing process descriptions are leveraged using natural language processing to further narrow down the search space for the mapping. Thereby, the manual effort to preprocess an event log for analysis is significantly reduced. The key contribution of this approach is the establishment of a relation between events and a given set of activities in a process model obtained by using (1) behavioral knowledge captured by Declare rules and (2) semantical knowledge entailed in labels and external process descriptions. As a result, mappings from events to activities can be obtained not only in a one-to-one fashion, but also in single-to-many relations. As shown by the conducted evaluation, the newly introduced matching technique performs well and requires little manual intervention. It also reveals to be robust toward noise.

## Footnotes

1
http://www.omg.org/.

2 In the literature, they are called “constraints”. Nevertheless, we prefer not to make use of such term, in order to avoid the conflict with “constraints” in the context of constraint satisfaction problems (CSPs).

3 Without loss of generality, loops can be unraveled and treated as an optional path that is traversable multiple times.

4 See http://lucene.apache.org.

5 See http://www.danielnaber.de/jwordsplitter/.

6 See http://processmining.org/prom/start.

7 The source code is available in the subversion repository at https://svn.win.tue.nl/repos/prom/Packages/Event2ActivityMatcher.

8 Fundamental Modeling Concepts (FMC) is a modeling notation where block diagrams are used to illustrate compositional structures as a composition of collaborating system components. For an introduction into FMC see [36].

9 See http://www-01.ibm.com/software/tivoli/products/ccmdb/features.html.
